# Alterations in rumen microbiota via oral fiber administration during early life in dairy cows

**DOI:** 10.1038/s41598-022-15155-0

**Published:** 2022-06-24

**Authors:** Heshan Kodithuwakku, Daiki Maruyama, Hisashi Owada, Yuto Watabe, Hiroto Miura, Yutaka Suzuki, Kazuo Hirano, Yasuo Kobayashi, Satoshi Koike

**Affiliations:** 1 Graduate School of Agriculture, Hokkaido University, Sapporo, 060-8589 Japan; 2 Central Research Institute for Feed and Livestock, ZEN-NOH, Kasama, 319-0205 Japan

**Keywords:** Microbial ecology, Bacterial host response, DNA sequencing

## Abstract

Bacterial colonization in the rumen of pre-weaned ruminants is important for their growth and post-weaning productivity. This study evaluated the effects of oral fiber administration during the pre-weaning period on the development of rumen microbiota from pre-weaning to the first lactation period. Twenty female calves were assigned to control and treatment groups (n = 10 each). Animals in both groups were reared using a standard feeding program throughout the experiment, except for oral fiber administration (50–100 g/day/animal) from 3 days of age until weaning for the treatment group. Rumen content was collected during the pre-weaning period, growing period, and after parturition. Amplicon sequencing of the 16S rRNA gene revealed that oral fiber administration facilitated the early establishment of mature rumen microbiota, including a relatively higher abundance of *Prevotella*, *Shuttleworthia*, *Mitsuokella*, and *Selenomonas*. The difference in the rumen microbial composition between the dietary groups was observed even 21 days after parturition, with a significantly higher average milk yield in the first 30 days of lactation. Therefore, oral fiber administration to calves during the pre-weaning period altered rumen microbiota, and its effect might be long-lasting until the first parturition.

## Introduction

The gastrointestinal tract of mammals is inhabited by a symbiotic microbial community, which influences the health and growth performance of the host animals^1^. The rumen represents a typical example of such environments, as feeds ingested by ruminants are degraded and fermented primarily by highly diverse microorganisms including bacteria, archaea, fungi, and protozoa into components such as short-chain fatty acids (SCFAs) and microbial proteins that can be used by the host^2^. Although the core bacterial members of rumen microbiota are widely distributed regardless of the breed or species of ruminants^3^, the relative abundance of each bacterial species in the rumen of individual animals varies extensively^4^. The relatively higher host specificity of the adult rumen microbiota is suggested by the quick reestablishment of the original bacterial community composition following a total exchange of the rumen contents^5^. Additionally, individual variations in rumen microbiota can influence host productivity^6^. Although the individuality of rumen microbiota can serve as a target for genetic selection and breeding^7^, it represents one of the hurdles in manipulating rumen microbiota via feeding management to improve host productivity. Yáñez-Ruiz et al.^8^ proposed the rumen programming concept, in which the composition of rumen microbiota is manipulated via feeding management during early life, leading to improved lifetime productivity. According to this concept, based on the immaturity of the rumen of pre-weaned calves in terms of function and microbiota^9^, rumen fermentation may be directed to the desired state by manipulating the establishment of microbiota via feeding intervention in the early life of calves.

The intake of solid feed, such as starter and roughage, increases in calves aged > 3 weeks and stimulates microbial proliferation^10^ and SCFA production, which facilitates rumen development^11^. Therefore, under normal feeding conditions, the morphological and functional development of the rumen is accelerated after 4–6 weeks of age^12^. The calf rumen is rapidly colonized by microbes immediately after birth^13^. Major fibrolytic bacterial species, such as *Fibrobacter succinogenes* and *Ruminococcus flavefaciens*, have been detected in calf rumen within the first 3 days after birth^14^. These observations suggest the solid feed can be potentially digested in the rumen of calves in their early life. Therefore, an increase in solid feed intake from the first week of life may stimulate microbes that have already colonized the rumen, leading to early rumen development.

Although several studies have indicated that increased roughage feeding to calves facilitates the colonization of rumen bacteria, including major cellulolytic species^15,16^, these studies have relied on the voluntary feed intake of calves. O’Hara et al.^9^ suggested that the rumen microbiota should be manipulated via early dietary intervention before 3 weeks of age, when rumen microbiota and voluntary intake of solid feed have not yet been established. Oral administration represents a feasible strategy to achieve an increased supply of solid feed before initiating voluntary intake. However, an appropriate method to achieve oral administration of solid feed to calves has not been developed yet. Therefore, we previously performed a feeding trial via oral fiber administration to newborn calves from 3 days after birth^17^ and confirmed that oral fiber administration improves body weight gain and gut microbiota in the first 3 weeks after birth. Since data on the effect of oral fiber administration on the health of neonatal calves were limited, the previous study mainly evaluated the presence of adverse effects of oral fiber administration on calf health, such as growth and gut environment. Thus, rumen microbiota in oral fiber-administered calves remained to be investigated.

The aim of the present study was to assess the effect of oral fiber administration from 3 days after birth on rumen bacterial colonization in pre-weaned calves and to evaluate its long-lasting effect on rumen microbiota in adult cows. We hypothesized that oral fiber administration before initiation of voluntary intake of solid diets could facilitate the early development of rumen microbiota via supplementation of growth substrates for bacteria. Based on the rumen programming concept, we presumed that dietary intervention from the early stage of life until weaning could impact the establishment of the rumen microbiota along with long-lasting effects on adult rumen microbiota. To test these hypotheses, we conducted long-term monitoring of rumen microbiota in individual dairy cows from the first week of life to 3 weeks after the first calving.

## Results

### Effect of oral fiber administration on rumen fermentation and structure of microbiota

A relatively higher butyrate proportion (*P* = 0.05) and lower ammonia nitrogen (NH_3_-N) concentration (*P* < 0.05) were detected in the rumen of the treatment group during the pre-weaning period, while significant differences were not detected in rumen fermentation parameters of adult animals throughout the prepartum and postpartum periods (Supplementary Table S1).

**Figure 1:**
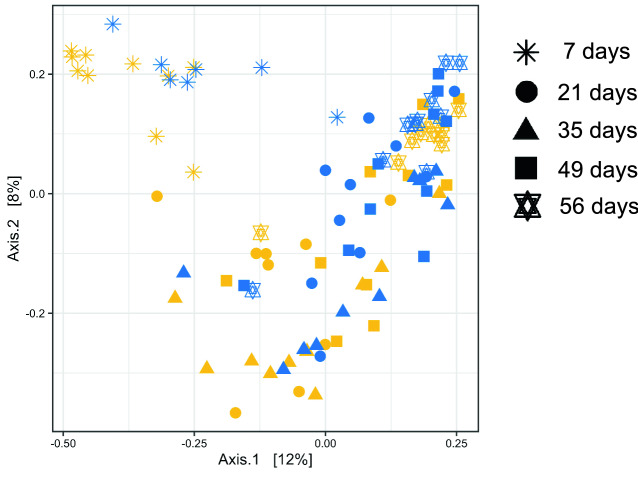
Change in the ruminal bacterial community structure in calves from 7 to 56 days of age. The PCoA plot was generated based on Bray–Curtis dissimilarities of rumen bacterial community determined via 16S rRNA gene amplicon sequencing. Colors indicate the dietary groups; control (yellow) and treatment (blue). Different symbols represent different age points. Individual points represent individual animals. *P*-values of the effect of dietary group (*P* < 0.001), calf age (*P* < 0.001), and diet × age interaction (*P* < 0.01) was calculated using the PERMANOVA test.

Amplicon sequencing generated a total of 3,701,563 non-chimeric reads, with 20,957 ± 346 reads/sample (mean ± standard error) for the control group and 21,110 ± 422 reads/sample (mean ± standard error) for the treatment group. After data processing for assignment of each read to bacterial taxa at the genus level, 11,216 unique amplicon sequence variants (ASVs) were detected. Principal coordinate analysis (PCoA) of the rumen bacterial community in calves (Fig. 1) showed that Bray–Curtis dissimilarities in the rumen bacterial community were affected by dietary group (*P* < 0.001), calf age (*P* < 0.001), and diet × age interaction (*P* < 0.01). Among the alpha diversity indices of the rumen microbiota in calves higher Shannon index (*P* < 0.05) was detected in the treatment group at 7 days of age (Supplementary Table S2). Chao1 and abundance-based coverage estimator (ACE) were affected (*P* < 0.05) by the dietary group in adult animals throughout the prepartum and postpartum periods (Supplementary Table S2).

### Alterations in the rumen microbiota of pre-weaned calves via oral fiber administration

**Figure 2:**
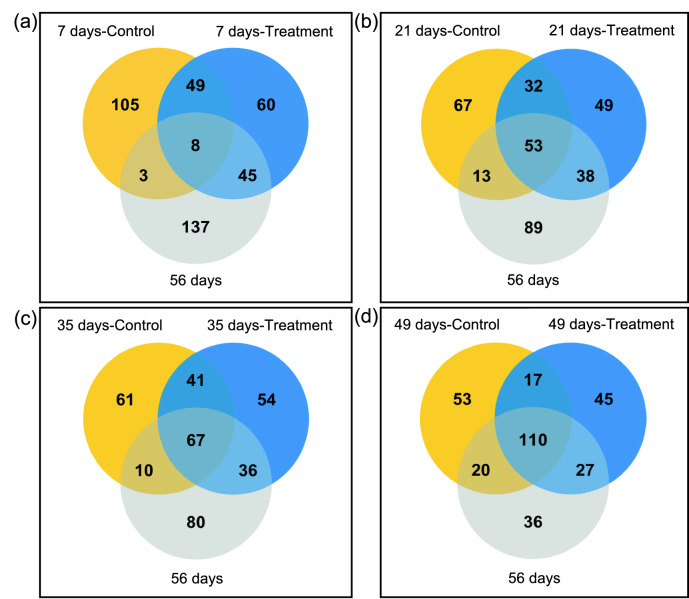
Bacterial ASVs shared by pre-weaned and weaned calves. Venn diagrams of ASVs shared by pre-weaned calves at (**a**) 7, (**b**) 21, (**c**) 35, and (**d**) 49 days of age and weaned calves at 56 days of age are shown. Pre-weaned calves were separated based on the dietary groups.

The bacterial communities clustered by the dietary group at 7–35 days of age (Supplementary Fig. S1). Of the 11,216 unique ASVs, 652 showed mean relative abundances of > 0.1% in either the control or the treatment groups at one of the ages (7, 21, 35, 49, or 56 days) and were used for the comparison (Fig. 2). Pre-weaned calves in the treatment group shared a larger number of bacterial ASVs with weaned calves than did those in the control group at 7 days of age (45 vs. 3 ASVs), and this trend continued throughout the pre-weaning period (38 vs. 13 ASVs at 21 days, 36 vs. 10 ASVs at 35 days, and 27 vs. 20 ASVs at 49 days of age).

**Figure 3:**
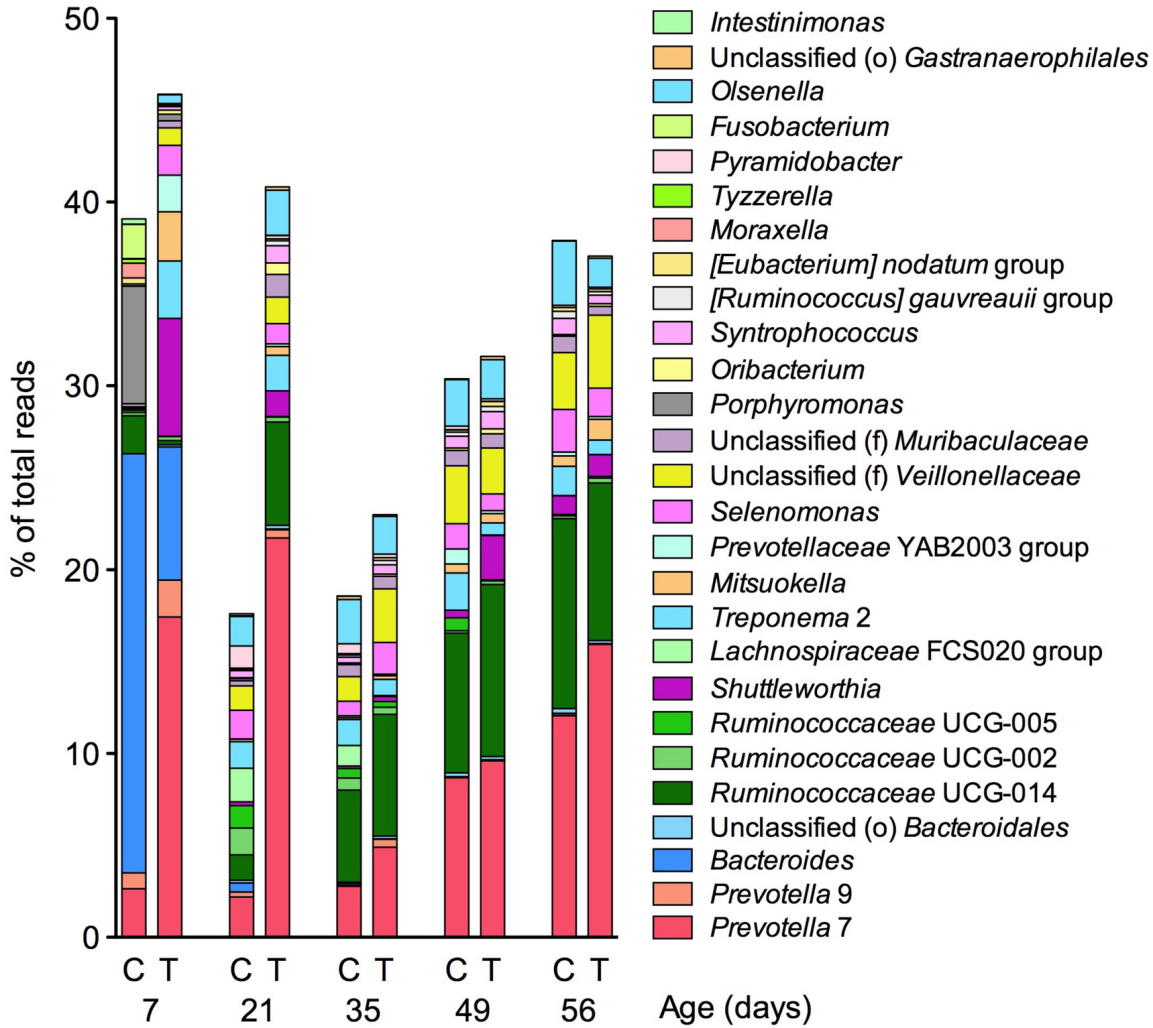
Change in the relative abundance of bacterial genera determined via amplicon sequencing. Bacterial genera showing > 0.1% relative abundances and significantly affected by the diet effect were selected from Supplementary Table S3, and the difference between the control and treatment groups at each sampling point were evaluated by a negative binomial model implemented as a Wald test in DESeq2 package in R software. Bacterial genera showing significant differences during at least one age point from 7 to 56 days of age are depicted in the graph. *C* control group, *T* treatment group.

Among the bacterial taxa detected via 16S ribosomal RNA (rRNA) gene amplicon sequencing on the rumen of calves aged 7–56 days (Supplementary Table S3), bacterial genera showing significant differences in their relative abundance by the treatment during at least one sampling point from 7 to 56 days of age were selected and are depicted in Fig. 3. We found remarkable differences in the rumen microbiota structure between the control and treatment groups within the first 21 days after birth (Fig. 3). At 7 days of age, 11 taxa showed a higher abundance in the treatment group, while 5 taxa showed lower abundance than that in the control group. Particularly, the abundance of *Prevotella* 7 (17.44% vs. 2.64%, *P* < 0.05), *Shuttleworthia* (6.41% vs. 0.09%, *P* < 0.01), *Mitsuokella* (2.67% vs. 0.02%, *P* < 0.01), and *Selenomonas* (1.60% vs. 0.01%, *P* < 0.01) was higher in the treatment group, while the abundance of *Porphyromonas* (0.36% vs. 6.41%, *P* < 0.05) was lower in the treatment group than that in the control group. Although significant difference was not observed, the abundance of *Bacteroides* was lower in the treatment group than that in the control group at 7 days of age. At 21 days of age, 9 taxa showed a higher abundance in the treatment group, whereas 6 taxa showed a lower abundance than that in the control group. Among them, the treatment group showed a higher abundance of *Prevotella* 7 (21.74% vs. 2.19%, *P* < 0.01), *Ruminococcaceae* UCG-014 (5.62% vs. 1.38%, *P* < 0.01), and *Shuttleworthia* (1.40% vs. 0.22%, *P* < 0.05) than that in the control group, while *Ruminococcaceae* UCG-005 (0.02% vs. 1.22%, *P* < 0.01), *Lachnospiraceae* FCS020 group (0.01% vs. 1.82%, *P* < 0.01), and *Bacteroides* (0.03% vs. 0.50%, *P* < 0.01) had a relatively lower abundance. No remarkable differences in abundance of the selected bacterial taxa were observed in animals aged 35–56 days.

### Follow-up monitoring of the rumen microbiota in the growing and parturition period

**Figure 4:**
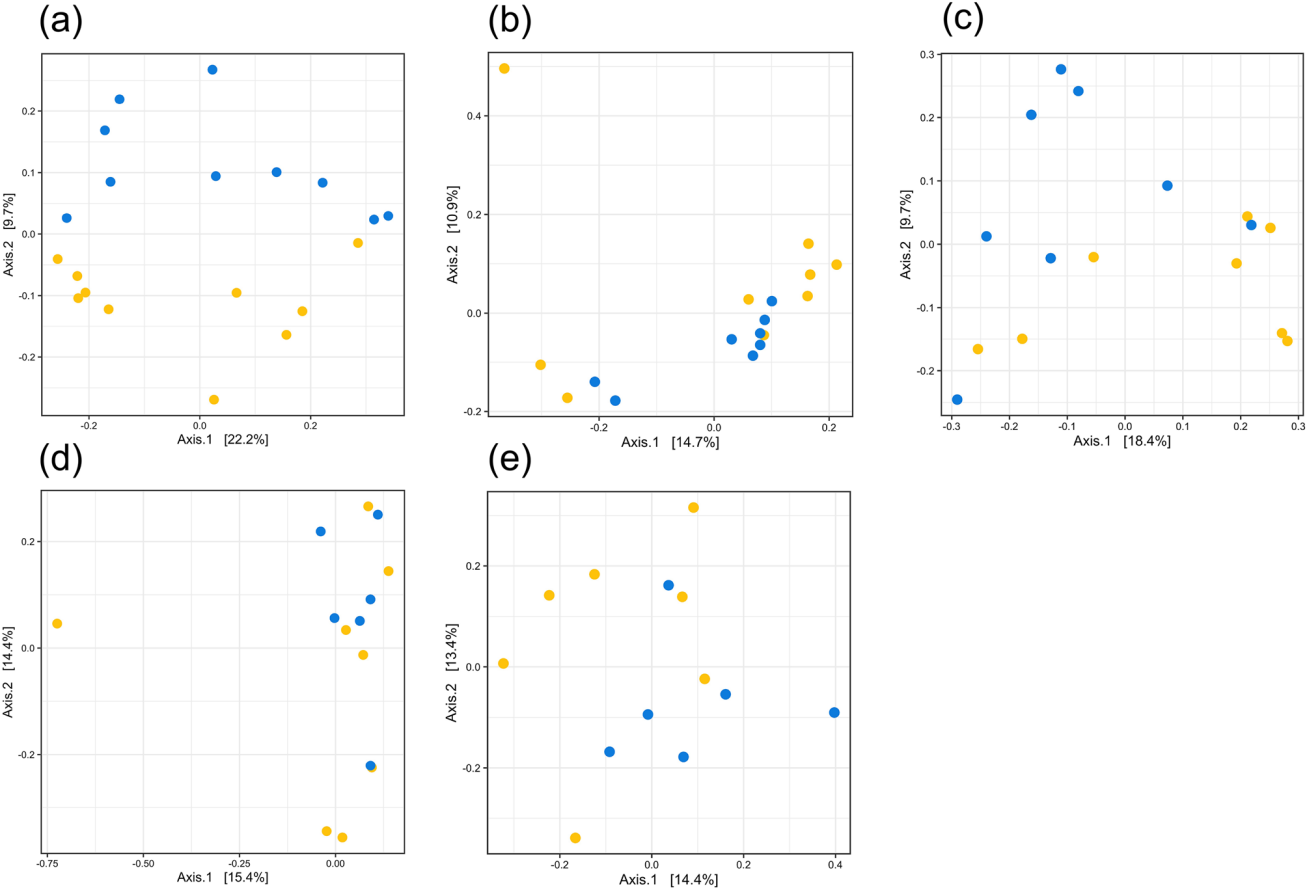
Change in the ruminal bacterial community structure in adult cows. The PCoA plot was generated based on Bray–Curtis dissimilarities in rumen bacterial communities determined via 16S rRNA gene amplicon sequencing for respective ages; (**a**) 9 months, (**b**) 60 days pre-calving, (**c**) 21 days pre-calving, (**d**) at calving, and (**e**) 21 days post-calving. Individual points in each plot represent individual animals. Colors indicate the dietary groups; control (yellow) and treatment (blue). *P*-values of the diet effect at 9 months (*P* = 0.02), 60 days pre-calving (*P* = 0.12), 21 days pre-calving (*P* = 0.05), at calving (*P* = 0.58), and 21 days post-calving (*P* = 0.03) were calculated using PERMANOVA test.

PCoA of the rumen bacterial community from 9 months of age until 21 days after parturition (Supplementary Fig. S2) showed that the beta diversity of the rumen microbiota was affected by diet (*P* = 0.01) and animal age (*P* < 0.001). The bacterial community structures differed by the dietary groups until 21 days after parturition (Fig. 4).

Among the bacterial taxa detected via 16S rRNA gene amplicon sequencing on the rumen microbiota in the growing and parturition period (Supplementary Table S4), bacterial genera that showed significant differences in relative abundance between the control and treatment groups at each sampling point are presented in Table 1. From 9 months of age to 21 days before calving, 4 taxa showed a higher abundance in the treatment group, whereas 5 taxa showed lower abundance than that in the control group. The treatment group had a lower abundance of *Prevotella* 1 (21.86% vs. 35.60%, *P* < 0.01) at 21 days before calving than that in the control group. The abundance of *Ruminiclostridium* 9 (0.45% vs. < 0.01%, *P* < 0.01) at calving and *Shuttleworthia* (0.52% vs. 0.11%, *P* < 0.01) and Unclassified (f) *Muribaculaceae* (0.10% vs. 0.00%, *P* < 0.01) at 21 days after calving were higher in the treatment group than that in the control group. Furthermore, these three bacterial taxa were differentially abundant in the treatment compared to control at 21 days after calving detected by the linear discriminatory analysis effect size (LEfSe) analysis (Supplementary Fig. S3).

**Table 1:** Bacterial taxa showing significant differences between the control and treatment groups at 9 months of age and around calving.

Bacterial taxa	Control	Treatment	*P*-value^a^	FDR^b^
**9 month of age**				
*Prevotellaceae* YAB2003 group	0.13 ± 0.03	0.02 ± 0.01	< 0.01	0.07
**21 days pre-calving**				
Uncultured (f) *Bacteroidales* BS11 gut group	0.36 ± 0.03	0.11 ± 0.03	< 0.01	0.02
*Prevotella* 1	35.60 ± 2.51	21.86 ± 3.21	< 0.01	0.08
*Prevotellaceae* NK3B31 group	0.08 ± 0.02	0.26 ± 0.06	0.01	0.15
*Acetitomaculum*	1.13 ± 0.10	2.22 ± 0.27	< 0.01	0.02
*Butyrivibrio* 2	1.74 ± 0.38	0.90 ± 0.05	< 0.01	0.07
*Eubacterium hallii* group	0.23 ± 0.03	0.54 ± 0.05	< 0.01	0.01
Uncultured (f) *Lachnospiraceae*	0.15 ± 0.02	0.07 ± 0.01	0.01	0.13
*Solobacterium*	0.09 ± 0.03	0.30 ± 0.04	< 0.01	0.02
**At calving**				
*Ruminiclostridium* 9	< 0.01 ± < 0.01	0.45 ± 0.31	< 0.01	0.07
**21 days post-calving**				
Unclassified (f) *Muribaculaceae*	0.00 ± 0.00	0.10 ± 0.05	< 0.01	0.11
*Shuttleworthia*	0.11 ± 0.02	0.52 ± 0.07	< 0.01	0.07

Values are presented as the mean ± standard error of relative abundance (percentage of total reads).

^a^*P*-values were calculated using the negative binomial model implemented as a Wald test in the DESeq2 package in R.

^b^False-discovery rate; statistical significance was determined using an FDR threshold of < 0.15.

**Figure 5:**
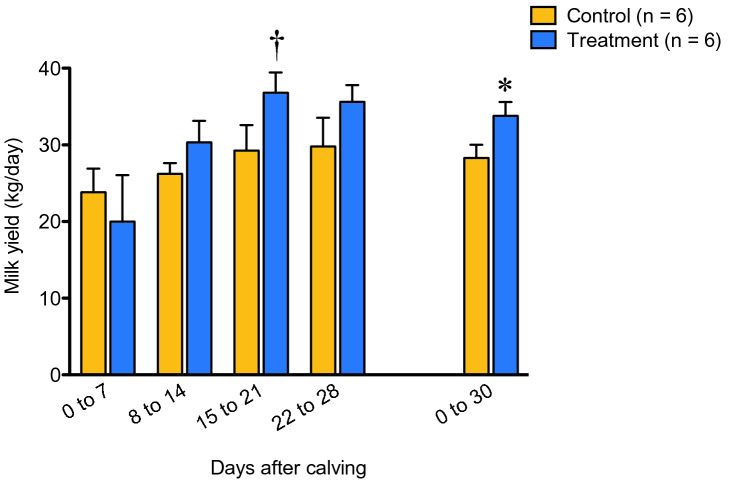
Average milk yield during the first 30 days of the lactation period. Milk yield was aggregated weekly or monthly, and *P*-values were calculated using the Welch’s *t*-test between the dietary groups at each time point. Asterisk (*) and dagger (†) indicate significance (*P* < 0.05) and tendency (*P* < 0.10), respectively.

**Figure 6:**
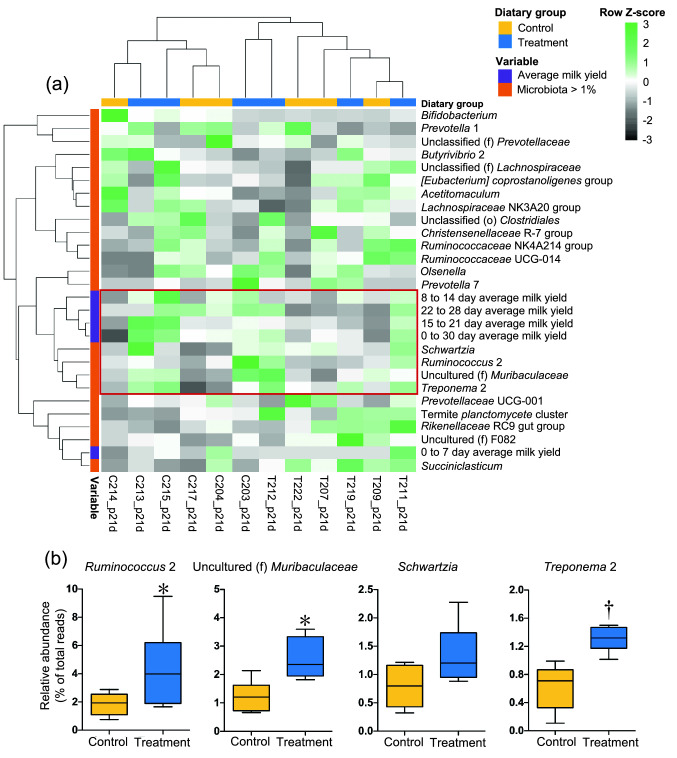
Exploration of the correlation between bacterial taxa and milk yield. (**a**) Heat map of hierarchical cluster analysis of rumen microbiota and milk yield. Rows represent the bacterial taxa (> 1.0% abundance) determined via 16S rRNA gene amplicon sequencing at 21 days after calving and average milk yield during the first 30 days of the lactation period. Columns represent the individual animals in each dietary group. Heat map color scale (black to green) displays the row Z-score (Z-score = [value of variable of an individual animal − mean of variable]/standard deviation). A dendrogram was generated based on Spearman’s correlation coefficient. (**b**) Relative abundance of the bacterial taxa clustered with milk yield in panel (**a**). Each box plot shows data of six animals and their range, median, and quartiles. Asterisk (*) and dagger (†) indicate significance (*P* < 0.05) and tendency (*P* < 0.10), respectively. *P*-values were calculated using a negative binomial model implemented as a Wald test in DESeq2 package in R software.

The body weights of the animals in both groups were not significantly different from 7 to 22 months of age (Supplementary Fig. S4). On the other hand, cows in the treatment group showed a higher (*P* < 0.05) average milk yield during the first 30 days of the first lactation period than that of cows in the control group (Fig. 5). Hierarchical cluster analysis of rumen microbiota at 21 days after calving and average milk yield during the first 30 days of lactation is shown in Fig. 6a. The genera *Ruminococcus* 2, Uncultured (f) *Muribaculaceae*, *Schwartzia*, and *Treponema* 2 were found to cluster together with the average milk yield of the cows. Furthermore, three of these four bacterial taxa were more abundant in the rumen of the treatment group at 21 days after calving than in the control group (Fig. 6b), and this observation was also confirmed by the LEfSe analysis (Supplementary Fig. S3).

## Discussion

The supplementation of roughage to calves during the pre-weaning period shows beneficial effects, such as improvement in feed intake, growth performance, rumination, and rumen environment^18,19^. We previously confirmed the beneficial effect of oral fiber administration on calves from 3 days of age to the age of weaning; calves that received oral fiber administration show improved body weight and hindgut microbiota within the first 3 weeks after birth^17^. The present study revealed that oral fiber administration to calves during the early stage of life altered rumen microbiota, and its effect might be long-lasting until the first parturition.

Diet plays a major role in ruminal microbial colonization in ruminants^3^. Pre-weaning diets primarily determine the nature of the rumen microbiota in calves^20^. In the present study, oral administration of a fibrous diet from 3 days of age resulted in the higher fibrous diet intake of calves until 28 days of age without affecting the starter intake (Supplementary Table S5). Therefore, supplementation of calves with a fibrous diet soon after birth leads to development of a distinct bacterial community in the rumen of pre-weaned calves. Pre-weaned calves in the treatment group shared a larger number of bacterial ASVs with weaned calves throughout the pre-weaning period in the present study, suggesting that oral fiber administration facilitates earlier colonization of rumen bacteria.

The distribution of *Prevotella* spp. within 21 days after birth was the most notable difference in the rumen microbiota of calves; oral fiber administration remarkably increased the abundance of *Prevotella* spp. This finding was further confirmed via real-time polymerase chain reaction (PCR) (Supplementary Table S6). *Prevotella* spp. are the most abundant genera in the rumen of mature ruminants^3^. When calves are fed primarily with milk or milk replacer during the first 14 days after birth, *Bacteroides* spp. dominate the rumen, and *Prevotella* spp. become dominant after 14 days of age, along with a gradual increase in solid feed intake^21^. In fact, the control group in the present study had a relatively higher abundance of *Bacteroides* spp. at 7 days of age, whereas the treatment group harbored *Prevotella* spp. at the same age. The present study indicates that early fibrous diet supplementation facilitates early colonization of the predominant rumen bacteria found in adult animals. The increase in abundance of *Prevotella* spp. via oral fiber administration is reasonable, since this genus represents one of the predominant plant cell wall-polysaccharide degraders found in the rumen^22^. *Prevotella* spp. utilize xylan by producing various enzymes for degradation^23^. The fibrous diet used in the present study contained Timothy hay and psyllium,the latter is mainly composed of heteroxylan. We previously observed that the abundance of *Prevotella* spp. is enhanced upon psyllium supplementation, but not by Timothy hay, in the feces of calves^17^. Therefore, psyllium supplementation may enhance the growth of *Prevotella* spp. in the rumen of calves within the first 21 d after birth.

Oral fiber-administered calves had a relatively higher abundance of *Shuttleworthia* spp.*, Mitsuokella* spp.*,* and *Selenomonas* spp.*,* with an increased proportion of butyrate at 7 days of age. *Shuttleworthia* spp. are known to produce butyrate^24^ which partly explains the relatively higher butyrate proportion observed at 7 days of age. *Mitsuokella* spp. produce lactate as the major fermentation product^25^, and *Selenomonas* spp. utilize lactate to produce propionate^26^. Although propionate proportion did not differ between the dietary groups at 7 days of age, the growth of these lactate-producing and lactate-utilizing bacteria may be enhanced in the rumen of calves upon oral fiber administration. Butyrate and propionate have been reported to enhance the development of the rumen epithelium^27,28^. Therefore, an increase in the abundance of bacterial taxa associated with butyrate and propionate production may be beneficial for calf growth by enhancing the development of the rumen epithelium.

Dietary interventions in early life can effectively manipulate rumen and fecal microbial communities, leading to an improvement in the early adult life productivity of ruminants^29,30^. In the present study, we observed the compositional difference in rumen microbiota and relatively higher average milk yield in the first 30 days of lactation period in cows that underwent early dietary intervention. Although feed intake record was not available due to total mixed ration feeding on the farm, the animals used in the present study were kept under the same feeding regimen after weaning. Therefore, we could assume that the early dietary intervention was the most significant factor affecting rumen microbiota establishment. Contrary to the present study, Dill-McFarland et al.^16^ reported that the supplementation of different solid diets during the pre-weaning period influences the early development of rumen microbiota in dairy calves without long-term effects on milk production. Although the discrepancies in findings between studies need to be clarified, the oral administration of a solid diet before initiation of voluntary intake may represent one of the factors leading to long-lasting effects.

In the present study, *Prevotella* was the predominant bacterial genus found in the rumen of both dietary groups during the prepartum and postpartum periods, which is in agreement with a previous study^31^. The treatment group had a relatively lower abundance of *Prevotella* 1 during the prepartum period. Similarly, real-time PCR quantification showed numerically lower abundance of *Prevotella* spp. in the treatment group during the prepartum period (Supplementary Table S7). The presence of this bacterial genus is negatively correlated with feed efficiency in bulls^32,33^. Therefore, a low abundance of *Prevotella* spp. in the treatment group may be beneficial during the prepartum period in terms of energy acquisition, as the nutritional status in the prepartum period influences milk yield and fertility parameters during lactation^34^.

Cluster analysis based on postpartum rumen microbiota composition and milk yield revealed a positive association of *Ruminococcus* 2, Uncultured (f) *Muribaculaceae*, *Schwartzia*, and *Treponema* 2 with milk production in the first 30 days of the lactation period. Tong et al.^35^ reported that *Ruminococcus* 2 and *Muribaculaceae* are relatively more abundant in dairy cows with higher milk yields. Another study reported that the abundance of *Treponema* and *Muribaculaceae* in the rumen is positively correlated with milk yield, milk fat content, milk protein content, and feed efficiency in dairy cows^36^. Furthermore, Cunha et al.^37^ suggested that the presence of *Schwartzia*, *Treponema,* and *Muribaculaceae* is negatively correlated with methane production in Holstein cows. Therefore, presence of these genera may contribute to efficient feed conversion into energy sources for milk production in dairy cows. Collectively, the relatively higher abundance of bacterial genera associated with efficient milk production may explain the relatively higher milk yield in the treatment group.

This study has several limitations that need to be clarified. Although we observed the compositional difference in rumen microbiota and relatively higher average milk yield in cows that underwent oral fiber administration during the early stages of life, the mechanism of action remains unknown. Furthermore, the rumen microbiota and the milk yield could also be affected by the genetic background of the animals. However, the host genetic influence was not controlled in the present study. In addition, the number of animals and experimental period were insufficient to evaluate the long-lasting effect of early dietary intervention on rumen microbiota and host productivity. This study monitored only the first month of lactation in a small number of animals. Further studies should evaluate relatively larger animal numbers and longer monitoring periods while minimizing the other factors (i.e., feed intake, host genetics) affecting rumen microbiota and milk yield. This will verify the possibility of manipulating the rumen microbiota in the early stages of life, which could contribute to improving host productivity.

In summary, we found that oral fiber administration from 3 days of age until weaning facilitated the early establishment of adult rumen microbiota in pre-weaned calves. This may explain the increased growth performance of oral fiber-administered calves in a previous study^17^. Furthermore, our dietary intervention strategy could exhibit a long-lasting effect on the rumen microbiota structure until 21 days after parturition, which may favorably affect milk production in dairy cows. Overall, our findings suggest that dietary intervention via oral fiber administration before initiation of voluntary intake of a solid diet alters rumen microbiota structure, which might contribute to improving host growth and productivity.

## Materials and methods

### Animals and diets

The animal experiments were conducted in accordance with the Guidelines for Animal Experiments and Act on Welfare and Management of Animals, Hokkaido University, and all experimental procedures were approved by the Animal Care and Use Committee of Hokkaido University. All animal experiments were carried out in accordance with ARRIVE guidelines. Twenty newborn female Holstein calves with an average birth weight of 37.1 ± 1.0 kg (mean ± standard error) were randomly allocated to either the control or treatment group at birth. All calves were housed individually in separate calf hutches containing sawdust bedding. Feeding and managing of animals until weaning at 50 d of age was performed as described previously^17^. After supplementing colostrum at birth, calves in both groups were fed 4 L of pasteurized whole milk (44.2% crude protein [CP] and 29.3% fat on a dry matter [DM] basis) as a transition milk during the first week since birth. From 8 days until weaning at 50 days of age, milk replacer (28.0% CP and 18.0% fat on a DM basis) was fed twice daily at 0830 and 1600 h. Water, calf starter (22.9% CP, 11.0% neutral detergent fiber [NDF], 5.6% acid detergent fiber [ADF], 6.2% crude ash, and 3.0% ether extract on a DM basis), and chopped Timothy hay (3.4% CP, 53.1% NDF, 34.2% ADF, 4.3% crude ash, and 1.7% ether extract on a DM basis) were provided for ad libitum intake from 3 days of age. In addition to voluntary intake of solid diets, the calves in the treatment group were orally administered with a mixture of ground Timothy hay and psyllium (4.4% CP, 78.6% NDF, 5.8% ADF, 3.9% crude ash, and 0.3% ether extract on a DM basis) from 3 days until weaning at 50 days of age. Timothy hay was ground for oral administration using a Wiley grinder (WM-3, Irie Shokai) with a 2-mm screen. To improve the handling of the treatment diet for oral administration, we incorporated psyllium, which is a dietary fiber that primarily improves gastrointestinal conditions in humans and can be incorporated in oral electrolyte solution supplemented to neonatal calves^38^. As a treatment diet, ground Timothy hay (50 g) and psyllium (6 g) were mixed with 200 mL of water. Owing to the adhesiveness of psyllium, the treatment diet formed a “hay ball” and showed slight stickiness, which facilitates swallowing by calves. At 3–7 days of age, one hay ball (50 g of fibrous diet) was orally administered after morning milk feeding. From 8 days of age to weaning, an additional hay ball was fed immediately after evening milk feeding (100 g fibrous diet per day).

After weaning, animals in both dietary groups were merged into the same herd and managed on the same farm under identical conditions. From 9 months of age until calving, heifers were fed a ration containing Timothy hay, alfalfa hay, fescue hay, and concentrate. After calving, the cows were fed a diet for lactating cows, as described in Supplementary Table S8. Diets comprised a total mixed ration and were fed twice daily at 0900 and 1600 h. All animals had ad libitum access to water and mineral blocks throughout the experiment. Daily milk production for each cow was measured for the first 30 days of the lactation period and the average values for each dietary group on a weekly and monthly basis were calculated. Milk yield for four animals in each dietary group were not recorded due to health problems including mastitis and displaced abomasum symptoms after calving.

In this study, all animals (n = 20) were maintained until 9 months of age, without severe problems. Owing to health problems, several animals were excluded from the experiment before parturition as follows: three animals (one in the control group and two in the treatment group) at 60 days before the expected calving date and one animal in the control group at 21 days before the expected calving date. One animal in the control group (15 days after calving) and two animals in the treatment group (calving day) were diagnosed with displaced abomasum symptoms and were excluded from further sampling. Owing to technical problems, samples were not collected from three animals aged 7 days in the treatment group and one animal aged 21 days in the control group. All other samples (n = 176) were obtained at the target sampling points.

### Sampling of rumen contents

Rumen contents were collected orally using a stomach tube. The stomach tube and the sample collection flask were thoroughly cleaned using water between sample collections from individual animals; the first fraction of the sample was discarded to avoid contamination from the previous sample and saliva. All samples were collected at 4 h after morning feeding. Rumen contents were collected at 7, 21, 35, 49, and 56 days, and at 9 months of age, 60 and 21 days before the expected calving date, at calving day, and 21 days after calving. The pH was measured using a pH meter (pH meter F-51; Horiba, Kyoto, Japan) immediately after sampling. Samples were collected in a sterile 50 mL tube and immediately placed on ice, followed by storage at − 30 °C until use.

### Chemical analysis

Rumen contents (1.0 g) were centrifuged at 16,000×*g* at 4 °C for 5 min, and the supernatant was collected. The SCFA content was analyzed using a gas chromatograph (GC-14B; Shimadzu, Kyoto, Japan) as described previously^39^. In brief, the supernatant of the rumen contents was mixed with 25% meta-phosphoric acid at a 5:1 ratio, incubated overnight at 4 °C, and centrifuged at 10,000×*g* at 4 °C. The supernatant was then mixed with crotonic acid as an internal standard and injected into a gas chromatograph equipped with an ULBON HR-20 M fused silica capillary column (0.53 mm i.d. × 30 m length, 3.0 µm film; Shinwa, Kyoto, Japan) and a flame-ionization detector. d/l-lactic acid levels were measured using a commercial assay kit (Megazyme International Ireland, Wicklow, Ireland) according to the manufacturer’s instructions. NH_3_-N levels were measured via the phenol-hypochloride reaction method^40^ using a microplate reader at 660 nm (ARVO MX; Perkin Elmer, Yokohama, Japan).

### DNA extraction and rumen microbiota profiling via amplicon sequencing

Total DNA was extracted and purified using the repeated bead-beating plus column method^41^. Rumen contents (0.25 g) were homogenized using sterile glass beads (0.4 g; 0.3 g of 0.1 mm and 0.1 g of 0.5 mm) and cell lysis buffer (1 mL; 500 mM NaCl, 50 mM Tris–HCl [pH 8.0], 50 mM ethylenediaminetetraacetic acid (EDTA), and 4% sodium dodecyl sulfate). The lysates were then incubated at 70 °C for 15 min, and the supernatant was collected for further processing. Bead-beating and incubation steps were repeated once, and all supernatants were combined. Total DNA was precipitated using 10 M ammonium acetate and isopropanol, followed by purification using the QIAamp Fast DNA Stool Mini Kit (Qiagen, Hilden, Germany). The DNA concentration was quantified using a Nanodrop 2000 spectrophotometer (Thermo Fisher Scientific, Waltham, MA, USA) and adjusted with Tris–EDTA buffer to the appropriate concentration.

For a comprehensive analysis of rumen bacterial communities, the MiSeq sequencing platform (Illumina, San Diego, CA, USA) was used. Total DNA obtained from the rumen contents was diluted to a final concentration of 5 ng/μL and subjected to PCR amplification of the V3-V4 regions of the 16S rRNA gene using the primer sets S-D-Bact-0341-b-S-17 (5′-CCTACGGGNGGCWGCAG-3′) and S-D-Bact-0785-a-A-21 (5′-GACTACHVGGGTATCTAATCC-3′)^42^. The PCR mixture consisted of 12.5 μL of 2× KAPA HiFi HotStart Ready Mix (Roche Sequencing, Basel, Switzerland), 0.1 μM of each primer, and 2.5 μL of DNA (5 ng/μL). PCR amplification was performed according to the following program described previously^9^: initial denaturation at 95 °C for 3 min; 25 cycles at 95 °C for 30 s, 55 °C for 30 s, and 72 °C for 30 s; and a final extension step at 72 °C for 5 min. Amplicons were purified using AMPure XP beads (Beckman-Coulter, Brea, CA, USA) and subjected to sequencing on the Illumina MiSeq platform (Illumina) using the MiSeq Reagent Kit v3 (2 × 300 paired-end). 

Data obtained from amplicon sequencing using the MiSeq platform were analyzed using QIIME2 version 2019.4^43^. Paired reads were filtered, dereplicated, merged, and chimera-filtered using the q2-dada2 plugin^44^ to generate ASVs. Taxonomic classification of the ASVs was performed at the phylum, class, order, family, and genus levels using the SILVA 132 99% operational taxonomic units, full length, seven level taxonomy classifier (silva-132-99-nb-classifier.qza). Sequenced data were processed further and analyzed using R software version 3.6.2^45^. ASV and taxonomy tables generated using QIIME2 were imported into R and merged with the sample metadata using the Phyloseq Bioconductor packages^46^. ASVs identified as Archaea, chloroplasts, and mitochondria were excluded. All samples were rarefied to a sampling depth of 16,805 reads, which was the smallest number of reads observed per sample in the filtered ASV table. Alpha diversity indices including Chao1, ACE, Shannon, and Simpson indices were calculated using the phyloseq function “estimate_richness”. PCoA was performed to determine differences in the microbial community structure based on the Bray–Curtis dissimilarity matrices at the genus level using the Phyloseq package. Venn diagrams were generated using ASVs showing mean relative sequence abundances of > 0.1% in either the control or the treatment groups at each sampling point. The relative abundance of each bacterial taxon was calculated by dividing the number of reads assigned to each taxon by the total number of reads. Taxa with an average relative abundance > 0.1% in > 50% of samples in either the control or treatment group during at least one sampling point were used for the analysis. Hierarchical cluster analysis of bacterial genera determined via amplicon sequencing at 21 days after calving and the weekly and monthly average milk yield for the first 30 days of lactation period was performed using the distances calculated from Spearman’s correlation and average linkage clustering.

### Quantification of target bacterial species/groups using real-time PCR

The relative abundance of known ruminal bacterial species and groups, including the total bacteria, *F. succinogenes*, *R. flavefaciens*, *Ruminococcus albus*, *Butyrivibrio* spp., *Prevotella* spp., *Selenomonas ruminantium*, *Megasphaera elsdenii*, *Treponema* spp., *Streptococcus bovis, Anaerovibrio lipolytica,* and *Ruminobacter amylophilus,* was quantified using real-time PCR. Amplification was performed using a Light Cycler 480 system (Roche Applied Science, Mannheim, Germany) with a KAPA SYBR Fast qPCR Kit (Roche Sequencing, Basel, Switzerland) and the respective primer sets (Supplementary Table S9). The standards used for the real-time PCR were prepared as described previously^47^. Briefly, plasmid DNA containing the respective target bacterial 16S rRNA gene sequence was obtained by PCR cloning using the species/genus-specific or bacterial universal primer sets. The concentration of the plasmid was determined with a spectrometer. Copy number of each standard plasmid was calculated using the molecular weight of nucleic acid and the length (base pair) of the cloned standard plasmid. Ten-fold dilution series ranging from 1 to 10^8^ copies were prepared for each target and run along with the samples. The respective genes were quantified using standard curves obtained from the amplification profile of the dilution series of the plasmid DNA standard (Supplementary Table S9). The PCR cycling conditions and reaction mixture were the same as those reported previously^48^. The relative abundance of each bacterial target was expressed as the proportion (%) of the abundance of the 16S rRNA genes of each bacterial target relative to that of the total bacteria.

### Statistical analysis

All data were sorted based on animal age into two sets, from 7 to 56 days of age and from 9 months of age to 21 days after calving, and analyzed separately. Data on fermentation parameters and bacterial abundance quantified via real-time PCR were analyzed using a repeated measures model using GraphPad Prism software version 9.1 (GraphPad Software, San Diego, CA, USA) with the fixed effects of dietary group, age, and diet × age interaction, and the random effect of animals within the groups. The Greenhouse–Geisser correction was used where sphericity was violated. If the *P*-value for the treatment effect was < 0.1, then Tukey’s method was used for multiple comparisons of the means. The effects of dietary group, age, and diet × age interaction on the bacterial taxon abundance determined via amplicon sequencing were assessed using the Poisson regression model in the lme4 package^49^ in R software. Dunn’s test was used for multiple comparisons, and *P*-values were adjusted using the Benjamini and Hochberg method^50^. Although the treatment effect was found to be significant for several bacterial genera, the post hoc test did not reveal any differences between the control and treatment groups at each sampling point (Supplementary Tables S3, S4). Therefore, the bacterial taxa determined via amplicon sequencing were further compared between the dietary groups at each sampling point using the negative binomial model implemented as the Wald test in the DESeq2 Bioconductor package in the R software^51^. A false discovery rate threshold of 0.15 was used to determine significance. Bray–Curtis dissimilarity matrices were calculated and visualized as PCoA using Phyloseq Bioconductor packages in R. permutational multivariate analysis of variance (PERMANOVA) tests were performed based on the Bray–Curtis dissimilarity matrices to assess the effect of dietary group, age, and diet × age interaction on microbial composition using the Vegan package version 2.5-7^52^ in R software.

## Supplementary Information

Supplementary Information.

## Data Availability

The raw sequences generated and analyzed during the study are available under BioProject accession number PRJNA715025 (https://www.ncbi.nlm.nih.gov/Traces/study/?acc=PRJNA715025) from the National Center for Biotechnology Information (NCBI) sequence read archive (SRA).
