# Supplementary material for: Alterations in rumen microbiota via oral fiber administration during early life in dairy cows
Source: Sci Rep. 2022 Jun 24;12:10798. doi: 10.1038/s41598-022-15155-0 (PMC9232566; doi:10.1038/s41598-022-15155-0)
Supplement: Supplementary file 1 — Supplementary Information. [file 41598_2022_15155_MOESM1_ESM.pdf]

Supplementary Information

Alterations in rumen microbiota via oral fiber administration during early life in dairy cows

Heshan Kodithuwakku[1], Daiki Maruyama[1], Hisashi Owada[2], Yuto Watabe[2], Hiroto Miura[1], Yutaka Suzuki[1], Kazuo Hirano[2], Yasuo Kobayashi[1], and Satoshi Koike[1]*

[1]Graduate School of Agriculture, Hokkaido University, Sapporo 060-8589, Japan

[2]Central Research Institute for Feed and Livestock, ZEN-NOH, Kasama 319-0205, Japan

Corresponding author*:

S. Koike

Graduate School of Agriculture, Hokkaido University,

Sapporo 060-8589, Japan

+81-(0)11-706-2812 (Phone & Fax)

skoike7@anim.agr.hokudai.ac.jp

Supplementary Table S1. Effect of oral fiber administration* on the rumen fermentation parameters in calves during 7 to 56 days of age and its long-lasting effect measured at 9 months of age and around calving

Item	Age (days)										SEM	*P*-value#		
	7		21		35		49		56					
	C	T	C	T	C	T	C	T	C	T		D	A	D × A
pH	6.84	6.20	6.14	6.22	6.30	6.17	6.22	6.09	5.93	6.02	0.10	0.24	0.01	0.10
Total VFA (mmol/L)	16.61	30.07	55.28	53.60	54.71	77.78	84.99	91.95	102.05	96.87	6.56	0.13	<0.01	0.06
Acetate (%)	67.51	62.82	57.80	53.16	57.16	52.02	50.84	51.57	49.52	51.22	1.54	0.07	<0.01	0.11
Propionate (%)	26.71	27.77	29.77	35.13	31.46	34.82	35.02	36.26	36.14	33.71	1.11	0.11	<0.01	0.10
Butyrate (%)	4.35^{b}	8.58^{a}	8.22^{ab}	7.87^{ab}	8.02^{ab}	9.40^{a}	9.35^{a}	8.30^{a}	10.10^{a}	11.09^{a}	0.61	0.05	<0.01	0.04
Acetate to propionate ratio	2.57	2.34	2.11	1.54	1.97	1.55	1.46	1.45	1.41	1.56	0.13	0.10	<0.01	0.18
NH_3-N (mg/dL)	8.38^{b}	7.27^{b}	23.09^{a}	13.28^{b}	14.73^{ab}	10.71^{b}	13.03^{b}	6.35^{b}	11.46^{b}	9.17^{b}	1.53	0.02	<0.01	0.35
Lactate (g/L)	0.14	0.34	0.29	0.17	0.27	0.20	0.36	0.55	0.40	0.38	0.06	0.55	0.06	0.33

Item	Age†										SEM	*P*-value		
	9 months		-60		-21		0		+21					
	C	T	C	T	C	T	C	T	C	T		D	A	D × A
pH	ND	ND	6.96	7.06	6.99	6.98	6.55	6.19	6.64	6.56	0.09	0.27	<0.01	0.14
Total VFA (mmol/L)	70.69	66.43	82.08	70.75	90.95	72.38	87.55	99.43	89.44	96.52	3.59	0.28	<0.01	0.01
Acetate (%)	66.69	67.70	66.71	67.18	65.18	66.18	61.35	61.24	60.81	59.99	0.77	0.54	<0.01	0.69
Propionate (%)	19.26	18.38	19.52	18.16	19.04	18.83	22.05	23.06	22.92	24.68	0.66	0.90	<0.01	0.26
Butyrate (%)	11.76	12.27	11.69	12.83	12.40	12.67	13.44	12.96	13.99	12.72	0.29	0.95	<0.01	0.03
Acetate to propionate ratio	3.50	3.72	3.44	3.71	3.43	3.53	2.89	2.71	2.67	2.45	0.13	0.67	<0.01	0.31
NH_3-N (mg/dL)	1.69	1.60	4.09	4.11	4.26	1.81	9.24	8.34	2.37	3.88	0.80	0.48	<0.01	0.27

Values are shown as mean.

C: control group.

T: treatment group.

ND: not determined.

*Calves in the treatment group were offered fibrous diet including timothy hay and psyllium (Kodithuwakku et al., 2021) via oral administration from 3 days of age until weaning in addition to the voluntary intake of timothy hay.

#*P*-values of the fixed effect of D (Dietary group), A (age) and D × A interaction was calculated by the repeated measure model.

†Days relative to calving except for 9 months of age.

a-bMean values with different superscripts differ ($P < 0.05$), for the treatment.

Supplementary Table S2. Effect of oral fiber administration[*] on the alpha diversity indices of the rumen microbiota in calves during 7 to 56 days of age and its long-lasting effect measured at 9 months of age and around calving

Item	Age (days)										SEM	*P*-value[#]		
	7		21		35		49		56					
	C	T	C	T	C	T	C	T	C	T		D	A	D × A
Chao1	127.94	175.07	217.18	210.32	221.06	228.91	233.08	260.90	228.30	250.50	5.44	0.08	<0.01	0.09
ACE[‡]	128.01	174.38	217.23	209.72	220.96	228.12	232.89	260.53	228.18	249.90	5.43	0.09	<0.01	0.09
Shannon	3.19^{c}	3.81^{b}	3.92^{b}	4.25^{ab}	4.12^{ab}	4.19^{ab}	4.24^{ab}	4.36^{a}	4.27^{ab}	4.40^{a}	0.05	0.04	<0.01	0.05
Simpson	0.89	0.94	0.94	0.97	0.95	0.95	0.97	0.95	0.97	0.97	0.005	0.29	<0.01	0.02

Item	Age[†]										SEM	*P*-value		
	9 months		-60		-21		0		+21					
	C	T	C	T	C	T	C	T	C	T		D	A	D × A
Chao1	539.88^{d}	583.78^{bcd}	667.14^{ac}	727.24^{a}	693.87^{ac}	709.60^{ab}	491.48^{d}	517.12^{d}	509.28^{d}	563.23^{cd}	12.74	0.03	<0.01	0.92
ACE	538.42^{d}	582.20^{bcd}	664.50^{ac}	723.62^{a}	692.29^{ac}	707.09^{ab}	490.50^{d}	514.70^{d}	508.30^{d}	562.35^{cd}	12.64	0.03	<0.01	0.92
Shannon	4.99	5.14	5.72	6.00	5.90	5.90	5.25	5.08	5.36	5.57	0.06	0.29	<0.01	0.61
Simpson	0.95	0.96	0.99	1.00	0.99	0.99	0.99	0.98	0.99	0.99	0.003	0.45	<0.01	0.50

Values are shown as mean.

C: control group.

T: treatment group.

[*]Calves in the treatment group were offered fibrous diet including timothy hay and psyllium (Kodithuwakku et al., 2021) via oral administration from 3 days of age until weaning in addition to the voluntary intake of timothy hay.

[#]*P*-values of the fixed effect of D (Dietary group), A (age) and D × A interaction was calculated by the repeated measure model.

[‡]ACE: abundance-based coverage estimator.

[†]Days relative to calving except for 9 months of age.

[a-d]Mean values with different superscripts differ ($P < 0.05$), for the treatment.

Supplementary Table S3. Effect of oral fiber administration[*] on the rumen microbiota in calves during 7 to 56 days of age determined by 16S rRNA gene amplicon sequencing at the genus level

Bacterial taxa	Age (days)										SEM	*P*-value[#]		
	7		21		35		49		56					
	C	T	C	T	C	T	C	T	C	T		D	A	D × A
Phylum: *Actinobacteria*														
Actinomyces	1.66	0.21	0.01	0.00	<0.01	0.00	0.00	0.00	0.00	0.00	0.07	1.00	<0.01	1.00
Bifidobacterium	0.17^{a}	0.36^{b}	0.04^{acd}	0.10^{bc}	0.02^{a}	0.03^{acd}	0.05^{ad}	0.06^{abcd}	0.07^{bcd}	0.05^{abcd}	0.02	0.04	<0.01	<0.01
Pseudoscardovia	0.06	0.03	0.04	0.05	<0.01	0.01	0.99	0.34	1.27	1.03	0.12	0.30	<0.01	<0.01
Olsenella	0.02^{a}	0.47^{ab}	1.62^{bc}	2.46^{c}	2.42^{bc}	2.04^{bc}	2.53^{bc}	2.12^{bc}	3.50^{c}	1.58^{bc}	0.22	0.01	<0.01	<0.01
Collinsella	0.19	0.22	0.02	0.00	0.00	0.00	0.00	0.00	0.00	0.00	0.01	1.00	<0.01	1.00
Phylum: *Bacteroidetes*														
Bacteroides	22.80^{a}	7.23^{a}	0.50^{ab}	0.03^{c}	0.11^{bc}	0.04^{bc}	0.02^{c}	0.02^{bc}	0.03^{bc}	0.01^{c}	0.88	0.03	<0.01	0.08
Uncultured (f) *Bacteroidales* UCG-001	0.00	0.01	0.03	0.01	0.34	0.10	0.02	0.02	<0.01	0.01	0.02	0.23	0.02	0.08
Uncultured (f) F082	0.00^{a}	0.00^{a}	0.43^{abc}	0.01^{ab}	0.72^{bc}	0.10^{abc}	0.77^{c}	0.65^{c}	0.36^{c}	0.42^{c}	0.09	<0.01	<0.01	<0.01
Butyricimonas	0.30	0.39	0.15	0.02	0.01	0.01	0.00	<0.01	0.00	<0.01	0.02	0.19	<0.01	<0.01
Uncultured (f) *Muribaculaceae*	0.00	0.01	0.00	0.05	1.37	0.32	1.08	0.81	0.00	0.18	0.14	0.90	<0.01	<0.01
**Unclassified (f) *Muribaculaceae***	0.18^{a}	0.38^{ab}	0.28^{ab}	1.23^{b}	0.66^{b}	0.68^{b}	0.83^{ab}	0.77^{ab}	0.89^{b}	0.48^{ab}	0.09	<0.01	<0.01	<0.01
Porphyromonas	6.41^{a}	0.36^{ab}	0.12^{abc}	0.01^{bcd}	0.03^{bcd}	0.00^{d}	<0.01^{cd}	0.00^{d}	0.00^{d}	<0.01^{cd}	0.31	0.01	<0.01	0.02
Alloprevotella	5.18	2.75	4.35	1.98	2.09	1.69	0.61	0.73	0.24	0.28	0.31	0.13	<0.01	<0.01
Prevotella 1	1.99	6.60	24.98	12.35	24.58	17.60	14.53	15.18	12.20	11.27	1.35	0.33	<0.01	0.08
***Prevotella* 7**	2.64^{ab}	17.44^{cd}	2.19^{ab}	21.74^{c}	2.77^{a}	4.90^{abd}	8.69^{abcd}	9.61^{bcd}	12.06^{cd}	15.94^{cd}	1.09	<0.01	<0.01	<0.01
***Prevotella* 9**	0.86^{a}	2.00^{b}	0.27^{ab}	0.44^{b}	0.06^{a}	0.43^{ab}	0.03^{a}	0.04^{ab}	0.11^{ab}	0.04^{ab}	0.14	0.03	<0.01	<0.01
Prevotellaceae UCG-001	0.17	0.29	0.79	1.27	2.01	1.30	1.90	1.47	0.63	1.47	0.14	0.95	<0.01	<0.01
Prevotellaceae UCG-004	<0.01	0.00	0.16	0.09	0.11	0.12	0.08	0.14	0.05	0.12	0.01	0.23	0.02	<0.01
***Prevotellaceae* YAB2003 group**	0.00^{a}	2.00^{b}	0.01^{ac}	0.14^{bc}	0.10^{abc}	0.09^{bc}	0.82^{b}	0.17^{bc}	0.20^{bc}	0.15^{b}	0.10	<0.01	<0.01	<0.01
Unclassified (f) *Prevotellaceae*	6.00^{abcd}	2.05^{abcd}	5.35^{abc}	5.45^{a}	4.38^{abcd}	4.98^{ac}	3.24^{abcd}	2.10^{bd}	1.56^{d}	2.19^{bcd}	0.33	0.03	<0.01	<0.01
Rikenellaceae RC9 gut group	0.01	0.28	2.33	1.11	3.64	2.58	1.49	2.02	1.10	1.97	0.30	0.12	<0.01	<0.01
Parabacteroides	0.59	0.26	0.12	0.00	<0.01	0.00	0.00	0.00	0.00	0.00	0.03	1.00	<0.01	1.00
uncultured *Rikenellaceae* bacterium	0.00	0.02	1.04	0.64	0.21	0.80	0.17	0.18	0.12	0.53	0.10	0.77	<0.01	<0.01
Uncultured (o) *Bacteroidales*	0.00^{a}	0.02^{ab}	2.00^{abc}	0.27^{bc}	1.17^{c}	1.00^{c}	0.49^{c}	0.54^{c}	0.28^{bc}	0.47^{bc}	0.15	<0.01	<0.01	<0.01

**Unclassified (o) *Bacteroidales***	<0.01^{a}	0.12^{abc}	0.14^{bc}	0.20^{b}	0.06^{ac}	0.15^{bc}	0.21^{bc}	0.18^{bc}	0.25^{b}	0.17^{bc}	0.01	<0.01	<0.01	<0.01
Phylum: *Cyanobacteria*														
Uncultured (o) *Gastranaerophilales*	0.00^{ab}	0.00^{ab}	0.00^{a}	0.00^{ab}	0.04^{ab}	0.01^{b}	0.03^{ab}	0.14^{ab}	0.01^{ab}	0.43^{ab}	0.04	<0.01	<0.01	<0.01
**Unclassified (o) *Gastranaerophilales***	0.00	<0.01	0.03	0.17	0.17	0.09	0.05	0.18	0.03	0.12	0.02	0.30	<0.01	0.54
Phylum: *Epsilonbacteraeota*														
Campylobacter	0.06	0.11	0.07	0.10	0.11	0.08	0.05	0.05	0.02	0.04	0.01	0.37	<0.01	0.01
Phylum: *Fibrobacteres*														
Fibrobacter	<0.01	0.03	0.42	0.80	1.14	0.64	0.69	0.18	0.22	0.06	0.09	0.19	<0.01	<0.01
Phylum: *Firmicutes*														
Enterococcus	0.50	1.52	0.00	0.00	0.00	0.00	0.00	<0.01	0.00	0.00	0.08	1.00	1.00	1.00
Lactobacillus	0.09^{a}	0.73^{ab}	0.03^{ab}	0.02^{ab}	0.03^{ab}	0.03^{ab}	0.10^{b}	0.16^{ab}	0.18^{b}	0.09^{b}	0.04	<0.01	<0.01	<0.01
Streptococcus	3.46^{a}	6.53^{a}	0.09^{ab}	0.04^{bc}	0.04^{bc}	0.03^{c}	0.01^{c}	0.01^{c}	0.02^{c}	<0.01^{c}	0.35	0.02	<0.01	<0.01
Christensenellaceae R-7 group	0.10	0.04	0.33	0.11	0.63	0.77	0.51	0.59	0.42	0.29	0.05	0.55	<0.01	<0.01
Unclassified (f) *Clostridiales* vadinBB60 group	0.01^{a}	0.30^{ab}	0.09^{b}	0.05^{ab}	0.04^{ab}	0.06^{ab}	0.02^{ab}	0.02^{ab}	0.02^{ab}	0.05^{ab}	0.02	<0.01	0.01	<0.01
Pseudoramibacter	<0.01^{a}	0.01^{ab}	0.03^{ab}	0.09^{bc}	0.05^{abc}	0.06^{abc}	0.16^{bc}	0.15^{c}	0.25^{c}	0.16^{c}	0.02	<0.01	<0.01	<0.01
Parvimonas	0.19	0.00	0.00	0.00	0.00	0.00	0.00	0.00	0.00	0.00	0.01	1.00	1.00	1.00
***[Eubacterium] nodatum* group**	0.32^{a}	0.03^{b}	0.05^{bc}	0.10^{abc}	0.07^{bc}	0.15^{ac}	0.13^{abc}	0.28^{a}	0.21^{a}	0.15^{a}	0.02	<0.01	<0.01	<0.01
Acetitomaculum	0.00^{a}	0.01^{ab}	0.06^{abc}	0.20^{c}	0.09^{bc}	0.10^{bc}	0.06^{bc}	0.06^{bc}	0.18^{bc}	0.04^{ab}	0.02	<0.01	<0.01	<0.01
Blautia	0.32	0.15	0.06	0.00	0.00	0.00	0.00	0.00	0.00	0.00	0.03	1.00	<0.01	1.00
Butyrivibrio 2	0.04	0.54	1.56	0.03	0.50	0.21	0.23	0.12	0.17	0.30	0.08	0.43	<0.01	<0.01
Catonella	0.00	0.00	0.02	0.03	0.12	0.16	0.18	0.13	0.03	0.03	0.02	0.68	<0.01	<0.01
Lachnoclostridium	0.48	0.07	0.03	0.00	0.01	0.00	0.00	0.00	0.00	0.00	0.02	1.00	<0.01	1.00
Lachnoclostridium 1	0.00	0.05	0.13	0.18	0.28	0.12	0.14	0.17	0.15	0.14	0.02	0.17	<0.01	<0.01
***Lachnospiraceae* FCS020 group**	<0.01^{a}	0.00^{a}	1.82^{b}	0.01^{a}	1.12^{ab}	0.06^{a}	<0.01^{a}	0.02^{a}	0.01^{a}	0.01^{a}	0.15	0.01	<0.01	<0.01
Lachnospiraceae NK3A20 group	0.14	2.63	6.72	7.15	6.57	8.70	7.02	6.66	5.10	7.53	0.60	0.16	<0.01	<0.01
Oribacterium	<0.01^{a}	0.23^{abc}	0.05^{ab}	0.62^{c}	0.06^{ab}	0.12^{abc}	0.13^{abc}	0.26^{bc}	0.09^{abc}	0.14^{ab}	0.04	<0.01	<0.01	<0.01
Pseudobutyrivibrio	0.00	0.00	0.08	0.05	0.50	0.15	0.19	0.29	0.16	0.18	0.03	1.00	<0.01	<0.01
Roseburia	1.28^{a}	0.26^{b}	0.35^{b}	0.29^{b}	0.44^{b}	0.18^{ab}	0.38^{b}	0.22^{ab}	0.22^{ab}	0.18^{ab}	0.12	0.01	<0.01	<0.01
Shuttleworthia	0.09^{a}	6.41^{b}	0.22^{ac}	1.40^{b}	0.13^{a}	0.27^{ac}	0.42^{abc}	2.42^{b}	1.01^{bc}	1.18^{b}	0.31	<0.01	<0.01	<0.01
Syntrophococcus	0.01^{a}	0.19^{ab}	0.38^{bcd}	0.94^{c}	0.31^{abd}	0.51^{bcd}	0.65^{bcd}	0.94^{bcd}	0.88^{cd}	0.47^{bcd}	0.06	<0.01	<0.01	<0.01

Tyzzerella	0.22^{a}	0.02^{ab}	0.00^{b}	0.00^{b}	0.00^{b}	0.00^{b}	0.01^{b}	0.01^{b}	<0.01^{b}	<0.01^{b}	0.01	<0.01	<0.01	<0.01
[Eubacterium] eligens group	0.25^{a}	0.79^{b}	0.07^{a}	0.24^{ab}	0.20^{a}	0.14^{a}	0.11^{a}	0.28^{ab}	0.15^{ab}	0.18^{ab}	0.03	<0.01	<0.01	<0.01
[Eubacterium] ruminantium group	0.00^{a}	0.84^{abc}	0.05^{abc}	0.11^{abc}	0.30^{abc}	0.05^{ab}	0.26^{abc}	0.23^{bc}	0.30^{bc}	0.72^{c}	0.05	<0.01	<0.01	<0.01
***[Ruminococcus] gauvreauii* group**	0.11^{a}	0.07^{ab}	0.08^{ab}	0.27^{bc}	0.12^{abc}	0.23^{bc}	0.21^{bc}	0.28^{c}	0.39^{c}	0.19^{bc}	0.02	<0.01	<0.01	<0.01
Unclassified (f) *Lachnospiraceae*	2.43	0.85	0.79	3.92	3.00	5.07	5.68	4.35	3.12	2.06	0.43	0.06	<0.01	<0.01
Intestinimonas	0.28^{a}	0.03^{bc}	0.11^{ab}	0.01^{c}	0.02^{c}	0.00^{c}	<0.01^{c}	0.00^{c}	0.00^{c}	0.00^{c}	0.02	<0.01	<0.01	0.18
Ruminococcaceae NK4A214 group	0.06	0.18	1.49	1.00	3.93	3.28	2.43	0.97	0.70	0.53	0.24	0.85	<0.01	<0.01
***Ruminococcaceae* UCG-002**	0.18^{a}	0.22^{ab}	1.46^{c}	0.27^{abc}	0.66^{bc}	0.38^{bc}	0.13^{ab}	0.19^{abc}	0.15^{ab}	0.27^{abc}	0.07	0.02	<0.01	<0.01
Ruminococcaceae UCG-004	0.17^{abc}	0.03^{a}	0.07^{abc}	0.07^{abc}	0.07^{abc}	0.05^{ab}	0.09^{abc}	0.14^{c}	0.13^{c}	0.10^{bc}	0.01	<0.01	<0.01	<0.01
***Ruminococcaceae* UCG-005**	0.12^{ab}	0.02^{a}	1.22^{bc}	0.02^{a}	0.52^{c}	0.31^{abc}	0.70^{abc}	0.05^{ab}	0.08^{abc}	0.08^{abc}	0.09	<0.01	<0.01	<0.01
Ruminococcaceae UCG-010	0.00	0.01	0.12	0.04	0.14	0.13	0.04	0.02	0.07	0.03	0.01	0.40	<0.01	1.00
Ruminococcaceae UCG-013	0.31^{a}	0.02^{ab}	0.04^{ab}	0.06^{abc}	0.05^{ab}	0.01^{a}	0.06^{abc}	0.16^{c}	0.06^{abc}	0.12^{bc}	0.03	<0.01	<0.01	<0.01
***Ruminococcaceae* UCG-014**	2.07^{a}	0.23^{a}	1.38^{ab}	5.62^{bc}	5.01^{bc}	6.62^{c}	7.61^{c}	9.36^{c}	10.32^{c}	8.55^{c}	0.54	<0.01	<0.01	<0.01
Ruminococcus 1	1.59^{a}	0.68^{a}	1.22^{ab}	1.23^{ab}	2.48^{c}	2.19^{bc}	1.58^{abc}	2.88^{c}	3.16^{bc}	2.52^{bc}	0.21	0.02	<0.01	<0.01
Ruminococcus 2	0.02	0.06	2.64	0.86	1.45	3.42	1.04	0.60	0.19	0.38	0.19	1.00	<0.01	<0.01
[Eubacterium] coprostanoligenes group	0.40	0.06	0.58	0.66	0.62	0.46	0.35	0.56	0.45	0.54	0.05	0.15	<0.01	<0.01
Unclassified (f) *Ruminococcaceae*	0.54^{a}	0.04^{a}	0.18^{ab}	0.11^{ab}	0.24^{b}	0.09^{ab}	0.14^{ab}	0.11^{ab}	0.09^{ab}	0.09^{ab}	0.05	<0.01	<0.01	<0.01
Catenisphaera	<0.01^{a}	0.02^{abc}	0.04^{abcd}	0.08^{bd}	0.01^{ac}	0.03^{abc}	0.06^{abcd}	0.11^{bcd}	0.18^{d}	0.16^{bd}	0.01	<0.01	<0.01	<0.01
Erysipelotrichaceae UCG-002	<0.01^{a}	<0.01^{ab}	0.03^{abc}	0.19^{abc}	0.07^{abc}	0.40^{cde}	0.59^{bcd}	1.79^{de}	1.92^{e}	2.39^{de}	0.15	<0.01	<0.01	<0.01
Erysipelotrichaceae UCG-006	0.02	0.03	0.17	0.14	0.10	0.06	0.17	0.47	0.42	0.34	0.04	0.81	<0.01	0.05
Erysipelotrichaceae UCG-009	0.17^{a}	0.42^{ab}	0.33^{ab}	0.66^{b}	0.20^{ab}	0.37^{ab}	0.56^{b}	0.69^{b}	0.59^{b}	0.47^{b}	0.05	<0.01	<0.01	<0.01
Faecalicoccus	0.37	0.42	0.03	0.00	<0.01	0.00	0.00	<0.01	0.00	0.00	0.03	0.10	<0.01	<0.01
Sharpea	0.04^{a}	0.31^{ab}	0.19^{b}	0.48^{b}	0.02^{a}	0.13^{ab}	0.48^{ab}	0.33^{b}	1.77^{b}	0.33^{b}	0.14	<0.01	<0.01	<0.01
Solobacterium	1.08	0.72	0.05	0.21	0.12	0.04	0.38	0.43	0.36	0.87	0.09	0.14	<0.01	<0.01
Uncultured (f)*Erysipelotrichaceae*	0.57^{a}	0.24^{bc}	0.16^{ab}	0.01^{bc}	0.01^{bc}	0.01^{c}	0.03^{abc}	0.02^{bc}	0.02^{bc}	0.01^{bc}	0.03	<0.01	<0.01	<0.01
Acidaminococcus	0.10^{a}	0.80^{ab}	0.55^{ab}	1.05^{bcd}	0.61^{ab}	0.85^{bc}	1.92^{bcde}	1.88^{cde}	2.90^{e}	2.22^{de}	0.14	<0.01	<0.01	<0.01
Phascolarctobacterium	1.09	0.80	0.24	0.00	<0.01	0.00	0.00	0.00	0.00	0.00	0.06	1.00	<0.01	1.00

Succiniclasticum	0.60	1.29	6.95	4.89	6.60	5.24	3.97	1.78	1.82	0.80	0.43	0.37	<0.01	<0.01
Dialister	0.05[a]	0.62[ab]	0.44[ab]	1.62[bcd]	0.44[ab]	1.67[bc]	3.41[cd]	3.71[cd]	3.98[d]	3.27[cd]	0.21	<0.01	<0.01	<0.01
Megasphaera	1.66[a]	2.85[ab]	3.04[ab]	2.77[ab]	1.10[a]	1.09[a]	3.00[ab]	2.26[ab]	6.33[b]	6.16[b]	0.36	0.02	<0.01	<0.01
Mitsuokella	0.02[a]	2.67[bcd]	0.13[abc]	0.48[bd]	0.10[ac]	0.20[abcd]	0.49[abcd]	0.51[bcd]	0.57[bcd]	1.13[d]	0.14	<0.01	<0.01	<0.01
Selenomonas	0.01[a]	1.60[ab]	1.56[b]	1.09[b]	0.78[ab]	1.70[b]	1.34[ab]	0.89[b]	2.31[b]	1.54[b]	0.17	<0.01	<0.01	<0.01
Veillonella	9.21	1.46	0.01	<0.01	<0.01	<0.01	0.01	0.00	0.00	0.00	0.49	0.80	<0.01	0.22
Veillonellaceae UCG-001	0.00	0.00	0.24	0.03	0.30	0.20	0.15	0.05	0.04	0.07	0.03	0.25	0.02	<0.01
Uncultured (f) *Veillonellaceae*	0.07	0.47	2.80	2.20	0.93	2.75	1.42	1.51	1.58	1.74	0.20	0.23	<0.01	<0.01
**Unclassified (f) *Veillonellaceae***	0.01[a]	0.97[ab]	1.33[b]	1.46[b]	1.35[b]	2.94[b]	3.17[b]	2.52[b]	3.11[b]	3.99[b]	0.25	<0.01	<0.01	<0.01
Phylum: *Fusobacteria*														
Fusobacterium	1.85[a]	0.01[b]	<0.01[b]	0.01[b]	<0.01[b]	<0.01[b]	0.00[b]	<0.01[b]	<0.01[b]	0.00[b]	0.09	<0.01	<0.01	<0.01
Phylum: *Patescibacteria*														
Candidatus Saccharibacteria bacterium UB2523	0.00[a]	<0.01[a]	0.00[a]	0.07[ab]	0.05[ab]	0.17[bcd]	0.06[abc]	1.71[d]	2.30[cd]	1.05[cd]	0.17	<0.01	<0.01	<0.01
Candidatus Saccharimonas	<0.01[a]	0.05[a]	0.08[a]	0.10[ab]	0.16[abc]	0.48[bcd]	0.71[cd]	0.76[d]	0.45[bcd]	0.70[cd]	0.06	0.02	<0.01	<0.01
Phylum: *Proteobacteria*														
Desulfovibrio	0.03	0.14	0.41	0.20	0.21	0.26	0.27	0.35	0.25	0.20	0.02	0.57	<0.01	0.07
Succinivibrio	0.04	0.50	2.53	0.59	0.67	0.69	0.94	0.37	0.34	0.54	0.15	0.35	<0.01	0.62
Succinivibrionaceae UCG-001	0.06	0.03	0.15	0.87	1.12	1.81	1.51	2.99	0.16	1.46	0.22	0.23	<0.01	<0.01
Comamonas	0.47	0.13	0.00	<0.01	<0.01	<0.01	0.01	0.03	0.01	0.01	0.04	0.22	<0.01	<0.01
Pelistega	0.55	0.83	0.01	0.00	0.01	0.00	0.00	0.00	0.00	0.00	0.06	1.00	<0.01	1.00
Neisseria	0.12[a]	0.02[ab]	0.01[bc]	<0.01[bc]	<0.01[bc]	<0.01[bc]	<0.01[bc]	0.00[c]	<0.01[bc]	0.00[c]	0.01	0.04	<0.01	1.00
Uncultured (f) *Neisseriaceae*	0.00	0.57	0.35	0.16	0.12	0.06	0.03	0.05	0.01	0.02	0.03	0.31	<0.01	<0.01
Bibersteinia	0.33[a]	0.06[a]	0.01[b]	<0.01[b]	0.00[b]	<0.01[b]	<0.01[b]	<0.01[b]	0.01[b]	0.00[b]	0.02	<0.01	<0.01	0.25
Gallibacterium	0.66	0.11	0.00	0.00	0.00	0.00	0.00	0.00	0.00	0.00	0.03	1.00	1.00	1.00
Unclassified (f) *Pasteurellaceae*	0.36	0.04	0.01	0.01	<0.01	0.00	<0.01	0.00	<0.01	0.00	0.02	0.09	<0.01	0.40
Moraxella	0.81[a]	0.03[ab]	0.01[bc]	<0.01[bc]	<0.01[bc]	<0.01[bc]	0.00[c]	<0.01[bc]	0.00[c]	0.00[c]	0.06	<0.01	<0.01	<0.01
Phylum: *Spirochaetes*														
Sphaerochaeta	0.04	0.10	0.53	0.44	0.27	0.19	0.09	0.11	0.11	0.10	0.02	0.42	<0.01	<0.01
Treponema 2	0.04[a]	3.13[ab]	1.45[b]	1.93[b]	1.42[b]	0.88[b]	2.02[b]	0.64[ab]	1.60[b]	0.79[ab]	0.21	<0.01	<0.01	<0.01
Phylum: *Synergistetes*														

Pyramidobacter	0.03^{a}	0.01^{a}	1.20^{b}	0.18^{bcd}	0.53^{bc}	0.19^{cd}	0.19^{bcd}	0.14^{acd}	0.11^{ad}	0.08^{ad}	0.05	<0.01	<0.01	<0.01
Phylum: *Tenericutes*														
Uncultured (o) *Mollicutes* RF39	0.00	0.00	0.00	0.00	0.06	0.10	0.11	0.24	0.08	0.15	0.02	0.30	<0.01	1.00
Unclassified (o) *Mollicutes* RF39	0.00	0.00	0.07	0.04	0.13	0.18	0.16	0.25	0.33	0.21	0.02	0.50	<0.01	0.03
Phylum: *Verrucomicrobia*														
Akkermansia	6.56	7.31	0.02	0.00	0.03	0.00	0.00	0.00	0.00	0.00	0.68	1.00	<0.01	1.00

Values are shown as the mean of relative abundance (% of total reads).

C: control group.

T: treatment group.

Bacterial genera in boldface showed a significant difference between the dietary groups by Wald test in DESeq2 package in R at least one of the sampling points and were selected for Figure 3.

[*]Calves in the treatment group were offered fibrous diet including timothy hay and psyllium (Kodithuwakku et al., 2021) via oral administration from 3 days of age until weaning in addition to the voluntary intake of timothy hay.

[#]*P*-values of the fixed effect of D (Dietary group), A (age) and D × A interaction was calculated by the Poisson regression model and adjusted using the Benjamini and Hochberg method.

[a-e]Mean values with different superscripts differ ($P < 0.05$), for the treatment.

Supplementary Table S4. Long-lasting effect of oral fiber administration[*] to calves on the rumen microbiota at 9 months of age and around calving determined by 16S rRNA gene amplicon sequencing at the genus level

Bacterial taxa	Age[†]										SEM	*P*-value[#]		
	9 months		-60		-21		0		+21					
	C	T	C	T	C	T	C	T	C	T		D	A	D × A
Phylum: *Actinobacteria*														
Bifidobacterium	0.00	0.00	0.00	0.00	0.00	0.01	0.22	0.04	2.78	0.80	0.15	0.26	<0.01	0.11
Uncultured (f) *Bifidobacteriaceae*	0.22	0.13	0.00	0.00	0.04	0.00	0.61	0.00	1.59	0.77	0.11	0.25	<0.01	<0.01
Atopobium	0.22^{a}	0.27^{ab}	0.19^{ab}	0.28^{c}	0.26^{a}	0.49^{abc}	0.33^{abc}	0.33^{abc}	0.35^{abc}	0.38^{bc}	0.02	0.01	<0.01	0.16
Olsenella	0.71	0.50	1.84	2.44	0.92	2.25	3.38	0.57	1.07	1.26	0.24	0.14	<0.01	<0.01
DNF00809	0.09^{b}	0.15^{ab}	0.22^{a}	0.23^{a}	0.14^{ab}	0.24^{ab}	0.28^{a}	0.27^{ab}	0.30^{a}	0.16^{ab}	0.02	<0.01	<0.01	<0.01
Enterorhabdus	0.03	0.05	0.05	0.08	0.03	0.15	<0.01	0.01	0.01	0.04	0.01	0.22	<0.01	<0.01
Phylum: *Bacteroidetes*														
**Uncultured (f) *Bacteroidales* BS11 gut group**	0.32^{de}	0.28^{ade}	0.51^{ade}	0.17^{abde}	0.36^{e}	0.11^{abcd}	0.06^{abc}	0.08^{abcd}	0.00^{c}	0.02^{bc}	0.04	0.05	<0.01	0.25
unidentified rumen bacterium RFN46	0.08	0.02	0.24	0.06	0.14	0.11	0.04	0.00	0.00	0.00	0.02	0.07	<0.01	0.01
Unclassified (f) *Bacteroidales* RF16 group	0.45^{b}	0.30^{ab}	0.41^{b}	0.25^{ab}	0.42^{b}	0.26^{ab}	0.16^{a}	0.57^{ab}	0.21^{ab}	0.15^{a}	0.04	<0.01	<0.01	<0.01
Uncultured (f) *Bacteroidales* UCG-001	0.06	0.05	0.25	0.22	0.24	0.24	0.03	0.04	0.03	0.05	0.02	0.67	<0.01	<0.01
Uncultured (f) F082	0.98	1.22	1.12	0.95	1.35	2.15	2.00	1.88	1.87	2.68	0.12	0.38	<0.01	0.14
Unclassified (f) F082	0.21	0.30	0.68	0.63	0.51	0.84	0.14	0.44	0.13	0.11	0.04	0.92	<0.01	<0.01
Uncultured (f) *Muribaculaceae*	0.64	1.02	0.59	0.73	0.94	1.80	1.14	2.51	1.09	2.56	0.10	0.14	<0.01	<0.01
**Unclassified (f) *Muribaculaceae***	0.03	0.09	0.02	0.03	0.18	0.12	0.16	0.27	0.00	0.10	0.02	0.35	<0.01	0.34
***Prevotella* 1**	28.74^{abc}	23.42^{bc}	33.93^{a}	31.37^{b}	35.60^{a}	21.86^{ac}	32.87^{a}	42.17^{a}	33.78^{a}	28.12^{abc}	1.23	<0.01	<0.01	<0.01
Prevotella 7	0.00^{c}	0.00^{c}	0.00^{c}	0.00^{c}	0.00^{c}	0.00^{c}	1.45^{ab}	0.04^{ac}	0.58^{ab}	1.99^{b}	0.17	<0.01	<0.01	<0.01
Prevotellaceae Ga6A1 group	0.02	0.04	0.04	0.06	0.07	0.06	0.24	0.30	0.06	0.20	0.02	0.39	<0.01	0.68
***Prevotellaceae* NK3B31 group**	0.02^{d}	0.03^{ad}	0.05^{acd}	0.14^{bc}	0.08^{abcd}	0.26^{b}	0.17^{abc}	0.17^{abcd}	0.34^{b}	0.50^{b}	0.02	<0.01	<0.01	<0.01
Prevotellaceae UCG-001	1.20	1.21	1.42	1.44	1.47	1.64	0.83	1.45	1.49	1.38	0.07	0.82	0.04	<0.01
Prevotellaceae UCG-003	0.70	1.24	1.70	1.63	1.10	1.50	0.56	1.57	0.45	0.39	0.10	0.06	<0.01	0.06
Prevotellaceae UCG-004	0.05	0.03	0.04	0.08	0.10	0.13	0.07	0.06	0.17	0.23	0.01	0.99	<0.01	0.06
***Prevotellaceae* YAB2003 group**	0.13^{a}	0.02^{bc}	0.00^{c}	0.01^{bc}	0.05^{abc}	0.01^{bc}	0.09^{ab}	0.19^{ab}	0.04^{abc}	0.12^{ab}	0.01	<0.01	<0.01	<0.01

Uncultured (f) *Prevotellaceae*	0.00	0.00	0.00	0.00	0.00	0.00	<0.01	0.00	0.09	0.14	0.01	0.92	<0.01	0.89
Unclassified (f) *Prevotellaceae*	0.53^{a}	0.82^{a}	0.54^{a}	0.76^{ab}	0.70^{a}	1.94^{ab}	2.97^{ab}	2.78^{ab}	2.89^{b}	2.70^{b}	0.25	0.02	<0.01	<0.01
Rikenellaceae RC9 gut group	3.59	4.22	5.31	5.60	6.67	7.63	4.00	2.83	3.88	3.88	0.22	0.10	<0.01	<0.01
Unclassified (f) *Rikenellaceae*	0.09	0.04	0.27	0.28	0.19	0.16	0.04	0.01	0.02	0.06	0.02	0.53	<0.01	0.09
Uncultured (f) p-251-o5	0.11	0.12	0.30	0.42	0.60	0.83	0.27	0.14	0.04	0.07	0.04	0.42	0.45	0.69
Uncultured (o) *Bacteroidales*	0.14	0.08	0.19	0.25	0.21	0.17	0.07	0.06	0.09	0.10	0.01	0.34	<0.01	0.05
Phylum: *Chloroflexi*														
Flexilinea	0.49^{adef}	0.63^{def}	0.39^{acdf}	0.52^{adef}	0.63^{ef}	0.87^{e}	0.35^{abcd}	0.19^{abc}	0.08^{b}	0.08^{bc}	0.04	0.01	<0.01	<0.01
Phylum: *Cyanobacteria*														
Uncultured (o) *Gastranaerophilales*	0.19^{cd}	0.08^{abc}	0.27^{d}	0.18^{cd}	0.14^{acd}	0.05^{abc}	0.03^{ab}	0.07^{abc}	0.01^{b}	0.05^{abc}	0.01	<0.01	<0.01	<0.01
Unclassified (o) *Gastranaerophilales*	0.07	0.03	0.08	0.10	0.11	0.16	0.01	0.03	0.01	0.06	0.01	0.12	<0.01	<0.01
Phylum: *Fibrobacteres*														
Fibrobacter	0.20	0.30	0.04	0.05	0.10	0.07	0.10	0.16	0.09	0.15	0.02	0.75	<0.01	<0.01
Phylum: *Firmicutes*														
Streptococcus	0.30	0.30	0.67	0.31	0.27	0.17	0.06	0.08	0.06	0.04	0.04	0.28	<0.01	0.85
Christensenellaceae R-7 group	3.65^{abc}	5.51^{c}	5.02^{c}	5.47^{c}	4.86^{c}	5.02^{bc}	2.50^{ab}	2.07^{a}	4.94^{bc}	3.81^{abc}	0.21	<0.01	<0.01	<0.01
Defluviitaleaceae UCG-011	0.04^{a}	0.08^{ab}	0.04^{a}	0.08^{ab}	0.08^{ab}	0.13^{ab}	0.16^{ab}	0.14^{ab}	0.11^{ab}	0.35^{b}	0.01	0.02	<0.01	0.85
Pseudoramibacter	0.08	0.10	0.04	0.02	0.02	0.04	0.30	0.04	0.19	0.19	0.02	0.38	<0.01	<0.01
Anaerovorax	0.06	0.09	0.11	0.13	0.14	0.20	0.05	0.07	0.07	0.11	0.01	0.39	0.53	0.16
Family XIII AD3011 group	0.31	0.35	0.68	0.60	0.65	1.01	0.83	0.70	0.58	0.50	0.04	0.19	<0.01	<0.01
[Eubacterium] nodatum group	0.08	0.07	0.16	0.13	0.13	0.19	0.26	0.10	0.29	0.25	0.02	0.82	<0.01	<0.01
Unclassified (f) Family XIII	0.02	0.03	0.04	0.03	0.05	0.08	0.14	0.14	0.20	0.20	0.01	0.10	<0.01	0.04
Acetitomaculum	0.64^{b}	0.91^{b}	0.92^{b}	1.11^{bc}	1.13^{bc}	2.22^{ac}	4.12^{a}	3.57^{a}	4.77^{a}	3.86^{a}	0.24	<0.01	<0.01	<0.01
Blautia	0.15	0.17	0.25	0.21	0.18	0.24	0.18	0.16	0.16	0.24	0.01	0.82	0.49	0.05
***Butyrivibrio* 2**	0.85^{ab}	0.53^{a}	1.96^{b}	1.27^{ab}	1.74^{b}	0.90^{ab}	1.08^{ab}	0.83^{ab}	1.12^{ab}	1.26^{ab}	0.09	<0.01	0.39	<0.01
Coprococcus 1	0.09	0.10	0.10	0.12	0.08	0.15	0.13	0.16	0.13	0.13	0.01	0.17	<0.01	0.50
Howardella	0.11^{ab}	0.06^{a}	0.11^{ab}	0.10^{ab}	0.10^{ab}	0.12^{ab}	0.13^{ab}	0.10^{ab}	0.17^{b}	0.16^{b}	0.01	0.05	<0.01	0.01
Lachnobacterium	0.00	0.00	0.00	0.00	0.00	0.00	0.17	0.16	0.02	0.01	0.01	0.54	<0.01	0.29
Lachnospira	0.01	0.00	0.04	0.04	0.00	0.00	0.45	0.21	0.07	0.22	0.03	0.20	<0.01	<0.01

Lachnospiraceae AC2044 group	0.05	0.07	0.11	0.17	0.19	0.15	0.07	0.09	0.01	0.04	0.01	0.68	0.03	0.85
Lachnospiraceae ND3007 group	0.27	0.41	0.31	0.21	0.19	0.33	0.19	0.16	0.15	0.18	0.03	0.09	<0.01	0.10
Lachnospiraceae NK3A20 group	1.79^{b}	2.08^{bc}	1.66^{bc}	2.07^{bcd}	2.03^{bc}	3.37^{acd}	7.47^{a}	5.85^{a}	7.91^{a}	4.66^{ad}	0.39	<0.01	<0.01	<0.01
Lachnospiraceae UCG-008	0.06	0.10	0.13	0.12	0.14	0.15	0.05	0.06	0.07	0.11	0.01	0.75	0.05	0.61
Lachnospiraceae XPB1014 group	0.57^{c}	0.43^{c}	0.59^{c}	0.41^{ac}	0.46^{c}	0.42^{c}	0.14^{ab}	0.28^{abc}	0.10^{b}	0.22^{abc}	0.03	<0.01	<0.01	<0.01
Marvinbryantia	0.02	0.04	0.05	0.11	0.04	0.13	0.09	0.21	0.16	0.37	0.02	0.07	<0.01	0.88
Pseudobutyrivibrio	0.34^{ab}	0.20^{a}	0.56^{b}	0.49^{b}	0.55^{b}	0.48^{b}	0.34^{ab}	0.32^{ab}	0.15^{a}	0.16^{a}	0.03	<0.01	<0.01	<0.01
Roseburia	0.01	0.01	0.06	0.10	0.06	0.09	0.18	0.07	0.09	0.17	0.01	0.34	<0.01	0.01
Shuttleworthia	0.00^{ab}	0.00^{a}	$<0.01^{b}$	$<0.01^{b}$	$<0.01^{b}$	0.00^{b}	0.59^{ab}	0.13^{ab}	0.11^{a}	0.52^{a}	0.06	<0.01	<0.01	<0.01
Syntrophococcus	0.03^{d}	0.10^{abd}	0.02^{d}	0.08^{ad}	0.04^{d}	0.10^{ad}	0.87^{abc}	0.11^{abcd}	0.94^{bc}	1.08^{c}	0.09	<0.01	<0.01	<0.01
[Eubacterium] cellulosolvens group	0.00^{b}	0.00^{b}	0.00^{b}	0.00^{b}	0.02^{b}	0.00^{b}	0.10^{a}	0.11^{a}	0.13^{a}	0.17^{a}	0.01	0.01	<0.01	<0.01
***[Eubacterium] hallii* group**	0.14	0.17	0.38	0.34	0.23	0.54	0.58	0.54	0.48	0.39	0.03	0.07	<0.01	<0.01
[Eubacterium] ruminantium group	0.21	0.14	0.18	0.21	0.38	0.22	0.67	0.22	0.22	0.41	0.04	0.22	<0.01	0.01
[Eubacterium] ventriosum group	0.13	0.23	0.30	0.27	0.27	0.29	0.09	0.13	<0.01	0.01	0.02	0.13	<0.01	0.43
[Ruminococcus] gauvreauii group	0.09	0.10	0.19	0.16	0.31	0.31	0.55	0.38	0.96	0.75	0.04	0.40	<0.01	<0.01
**Uncultured (f) *Lachnospiraceae***	0.07^{ab}	0.03^{a}	0.09^{abc}	0.07^{abc}	0.15^{c}	0.07^{abc}	0.06^{ab}	0.10^{abc}	0.08^{abc}	0.16^{bc}	0.01	<0.01	0.23	<0.01
Unclassified (f) *Lachnospiraceae*	1.39	1.34	1.30	1.35	1.30	1.54	1.61	1.41	1.57	1.54	0.06	0.84	<0.01	0.21
Papillibacter	0.09	0.13	0.30	0.38	0.43	0.55	0.02	0.01	0.01	<0.01	0.02	0.74	<0.01	0.40
Ruminiclostridium	0.24	0.24	0.20	0.18	0.14	0.29	0.09	0.10	0.20	0.15	0.02	0.99	<0.01	0.02
Ruminiclostridium 6	0.13	0.11	0.06	0.09	0.05	0.02	0.07	0.02	0.01	0.03	0.01	0.45	<0.01	<0.01
***Ruminiclostridium* 9**	0.71^{b}	0.34^{bc}	1.60^{b}	0.45^{b}	0.59^{b}	0.56^{bc}	$<0.01^{a}$	0.45^{abc}	0.00^{a}	0.05^{ac}	0.09	0.01	<0.01	<0.01
Ruminococcaceae NK4A214 group	4.52^{ab}	7.23^{b}	4.84^{ab}	5.84^{ab}	4.66^{ab}	5.31^{ab}	3.29^{a}	3.09^{a}	3.83^{a}	4.46^{ab}	0.22	<0.01	<0.01	<0.01
Ruminococcaceae UCG-001	0.22	0.28	0.03	0.12	0.05	0.09	0.07	0.06	0.09	0.08	0.03	0.29	<0.01	<0.01
Ruminococcaceae UCG-002	0.11	0.14	0.15	0.13	0.13	0.14	0.00	0.01	0.02	0.11	0.01	0.39	<0.01	<0.01
Ruminococcaceae UCG-005	0.16	0.23	0.32	0.52	0.58	1.03	0.13	0.13	0.02	0.16	0.04	0.39	<0.01	<0.01
Ruminococcaceae UCG-010	0.12	0.14	0.62	0.55	0.61	0.71	0.11	0.13	0.07	0.11	0.04	0.75	<0.01	<0.01
Ruminococcaceae UCG-011	0.00^{a}	0.01^{ab}	0.02^{ab}	0.10^{c}	0.06^{bc}	0.09^{abc}	0.03^{abc}	0.02^{abc}	0.00^{ab}	0.00^{ab}	0.01	<0.01	<0.01	<0.01

Ruminococcaceae UCG-013	0.14	0.22	0.32	0.30	0.15	0.13	0.03	0.05	0.02	0.19	0.02	0.92	<0.01	<0.01
Ruminococcaceae UCG-014	0.55^{a}	0.98^{abc}	0.65^{ab}	0.93^{abc}	0.61^{ab}	0.98^{abc}	0.89^{abc}	1.39^{abc}	1.87^{bc}	2.34^{c}	0.09	<0.01	<0.01	<0.01
Ruminococcaceae V9D2013 group	0.56	0.51	0.12	0.34	0.02	0.02	0.01	0.00	0.00	0.00	0.04	0.54	<0.01	<0.01
Ruminococcus 1	0.78	0.91	0.34	0.46	0.37	0.40	0.34	0.22	0.22	0.44	0.04	0.45	<0.01	0.11
Ruminococcus 2	2.62	3.30	1.45	1.67	1.23	2.11	3.31	3.39	1.76	4.37	0.22	0.37	<0.01	<0.01
Saccharofermentans	0.41	0.60	0.73	0.87	1.13	0.95	0.49	0.49	0.47	0.78	0.04	0.18	<0.01	0.06
[Eubacterium] coprostanoligenes group	0.43^{b}	0.62^{ab}	0.74^{ab}	0.88^{ab}	0.76^{ab}	1.11^{a}	1.15^{a}	0.66^{ab}	1.13^{a}	1.16^{a}	0.05	<0.01	<0.01	<0.01
Uncultured (f) *Ruminococcaceae*	0.87	0.51	0.21	0.20	0.24	0.54	0.19	0.08	0.16	0.16	0.05	0.07	<0.01	<0.01
Unclassified (f) *Ruminococcaceae*	0.29	0.17	0.26	0.34	0.25	0.26	0.11	0.02	0.07	0.10	0.02	0.19	<0.01	<0.01
Unclassified (o) *Clostridiales*	2.81	3.63	4.43	4.67	3.93	4.39	1.44	1.43	1.00	1.07	0.18	0.20	<0.01	<0.01
Erysipelotrichaceae UCG-004	0.18	0.18	0.47	0.47	0.38	0.81	0.41	0.31	0.28	0.30	0.05	0.46	<0.01	0.10
Erysipelotrichaceae UCG-009	0.15	0.17	0.29	0.28	0.28	0.32	0.46	0.24	0.54	0.36	0.02	0.07	<0.01	<0.01
Kandleria	0.01	<0.01	<0.01	<0.01	0.10	0.07	0.62	0.09	0.33	0.15	0.06	0.82	<0.01	0.30
Sharpea	0.00	0.00	0.00	0.00	0.00	0.00	0.16	0.00	0.02	0.69	0.03	0.75	<0.01	0.67
Solobacterium	0.10^{a}	0.16^{ab}	0.22^{ab}	0.28^{b}	0.09^{a}	0.30^{b}	0.18^{ab}	0.25^{ab}	0.26^{ab}	0.17^{ab}	0.02	<0.01	<0.01	<0.01
[Anaerorhabdus] furcosa group	0.06^{a}	0.14^{ab}	0.08^{ab}	0.07^{ab}	0.08^{ab}	0.15^{ab}	0.10^{ab}	0.14^{ab}	0.16^{ab}	0.19^{b}	0.01	<0.01	<0.01	0.03
Uncultured (f) *Erysipelotrichaceae*	0.06^{a}	0.10^{ab}	0.09^{ab}	0.09^{ab}	0.11^{ab}	0.09^{ab}	0.09^{a}	0.05^{a}	0.18^{b}	0.18^{b}	0.01	0.03	<0.01	<0.01
Succiniclasticum	2.39	2.84	1.77	2.39	1.79	1.97	1.01	0.94	1.36	1.51	0.11	0.10	<0.01	<0.01
Anaerovibrio	0.05	0.03	0.02	0.03	0.04	0.02	0.21	0.15	0.07	0.10	0.01	0.26	<0.01	0.03
Quinella	20.01	15.32	0.50	0.15	0.16	0.16	<0.01	0.01	0.00	0.00	1.08	0.60	<0.01	<0.01
Schwartzia	0.17	0.14	0.16	0.16	0.23	0.23	0.26	0.32	0.89	1.35	0.05	0.12	<0.01	<0.01
Selenomonas 1	0.86	0.74	0.46	0.53	0.54	0.25	0.16	0.54	0.31	0.40	0.05	0.22	<0.01	<0.01
Veillonellaceae UCG-001	0.55	0.52	0.55	0.55	0.62	0.53	0.48	0.85	0.21	0.43	0.03	0.09	<0.01	<0.01
Uncultured (f) *Veillonellaceae*	0.28	0.28	0.38	0.51	0.32	0.25	0.16	0.27	0.05	0.00	0.03	0.96	<0.01	0.11
Unclassified (f) *Veillonellaceae*	0.18	0.08	0.14	0.20	0.12	0.05	0.04	0.04	0.05	0.11	0.01	0.07	<0.01	<0.01
Phylum: *Kiritimatiellaeota*														
Uncultured. (o) WCHB1-41	1.25	1.48	1.73	1.26	2.09	2.12	0.52	0.62	0.08	0.09	0.12	0.76	<0.01	0.30
Unclass. (o) WCHB1-41	0.06	0.16	0.08	0.11	0.10	0.11	0.01	0.01	0.01	0.02	0.01	0.10	<0.01	<0.01

Phylum: *Lentisphaera*														
Uncultured (f) vadinBE97	0.10	0.09	0.20	0.23	0.17	0.10	0.01	0.02	0.00	0.00	0.01	0.82	<0.01	0.85
unidentified rumen bacterium RFN4	0.15	0.10	0.22	0.21	0.24	0.17	0.01	0.04	<0.01	<0.01	0.02	0.38	<0.01	0.11
Phylum: *Patescibacteria*														
Uncultured (o) *Absconditabacteriales (SR1)*	0.04	0.07	0.05	0.13	0.04	0.10	0.01	0.02	0.01	0.03	0.01	0.07	<0.01	0.32
Candidatus Saccharimonas	0.86	0.99	1.21	1.31	0.96	1.05	0.44	0.51	0.60	0.63	0.05	0.40	<0.01	0.26
Phylum: *Planctomycetes*														
CPla-4 termite group	0.45	0.79	1.18	1.29	1.14	1.30	0.05	0.10	0.13	0.63	0.09	0.72	<0.01	<0.01
Pirellula	0.07	0.10	0.13	0.23	0.07	0.08	0.03	0.09	0.11	0.03	0.01	0.10	0.03	<0.01
Termite *planctomycete* cluster	0.14	0.23	0.15	0.11	0.08	0.03	0.29	0.43	0.45	0.75	0.04	0.99	<0.01	0.04
p-1088-a5 gut group	0.95^{ab}	1.73^{d}	1.30^{bcd}	1.34^{cd}	1.39^{abcd}	1.48^{abcd}	0.87^{a}	1.04^{abc}	0.91^{ab}	0.97^{abc}	0.07	<0.01	<0.01	<0.01
Phylum: *Spirochaetes*														
Treponema 2	0.35^{acd}	0.21^{cd}	0.23^{cd}	0.24^{cd}	0.24^{cd}	0.15^{d}	1.00^{abc}	1.19^{ab}	0.56^{abc}	1.31^{b}	0.06	<0.01	<0.01	<0.01
Phylum: *Synergistetes*														
Fretibacterium	0.04	0.01	0.05	0.12	0.02	0.01	0.01	0.01	0.01	0.01	0.01	0.70	<0.01	0.35
Phylum: *Tenericutes*														
Anaeroplasma	0.16	0.23	0.37	0.40	0.14	0.15	0.20	0.20	0.20	0.28	0.02	0.16	0.71	0.29
Unclassified (o) *Mollicutes* RF39	0.07	0.09	0.09	0.08	0.06	0.07	0.10	0.16	0.10	0.10	0.01	0.75	0.01	0.47
Phylum: *Verrucomicrobia*														
Uncultured (o) LD1-PB3	0.50	0.14	0.18	0.40	0.33	0.19	0.06	0.06	0.00	0.00	0.04	0.44	<0.01	<0.01
Uncultured (f) *Pedosphaeraceae*	0.17	0.08	0.33	0.16	0.24	0.12	0.01	0.03	0.00	0.00	0.03	0.09	<0.01	0.04

Values are shown as the mean of relative abundance (% of total reads).

C: control group.

T: treatment group.

Bacterial genera in boldface showed a significant difference between the dietary groups by Wald test in DESeq2 package in R at least one of the sampling points and were selected for Table 1.

*Calves in the treatment group were offered fibrous diet including timothy hay and psyllium (Kodithuwakku et al., 2021) via oral administration from 3 days of age until weaning in addition to the voluntary intake of timothy hay.

†Days relative to calving except for 9 months of age.

#*P*-values of the fixed effect of D (Dietary group), A (age) and D × A interaction was calculated by the Poisson regression model and adjusted using the Benjamini and Hochberg method.

a-fMean values with different superscripts differ ($P < 0.05$), for the treatment.

Supplementary Table S5. Feed intake of calves from birth to 56 days of age.

Age (days)	Milk[1] (kg DM/day)	Fibrous diet[2] (kg DM/day)				Starter (kg DM/day)			
		Control	Treatment	SEM	*P*-value[3]	Control	Treatment	SEM	*P*-value
1 to 7	0.55	< 0.01	0.03	< 0.01	< 0.01	0.01	0.02	< 0.01	0.58
8 to 14	0.76	< 0.01	0.10	0.01	< 0.01	0.03	0.05	0.01	0.18
15 to 21	0.96	0.04	0.10	0.01	< 0.01	0.08	0.08	0.01	0.82
22 to 28	0.96	0.10	0.14	0.01	0.03	0.16	0.20	0.03	0.54
29 to 35	0.96	0.14	0.18	0.01	0.09	0.21	0.28	0.03	0.29
36 to 42	0.57	0.27	0.29	0.01	0.36	0.52	0.62	0.06	0.36
43 to 49	0.38	0.34	0.39	0.02	0.25	0.92	0.92	0.06	0.99
50 to 56	nil	0.53	0.58	0.03	0.35	1.28	1.20	0.05	0.37

Values are shown as mean (n=10).

[1]Both groups were fed the same amounts of milk (transition milk during 1 to 7 day, milk replacer from 8 to 49 day, and weaned after 49 days of age) by feeding bottle as shown in each time points.

[2]Total fibrous diet intake (oral administration of timothy hay and psyllium + voluntary intake of timothy hay). Calves in the treatment group were subjected to oral administration of timothy hay and psyllium from 3 days until weaning at 50 days of age.

[3]*P*-values were calculated by student's *t*-test.

Supplementary Table S6. Effect of oral fiber administration* on the major rumen bacterial species/groups in calves during 7 to 56 days of age quantified by quantitative real-time PCR

Bacterial species/group	Age (days)										SEM	*P*-value#		
	7		21		35		49		56					
	C	T	C	T	C	T	C	T	C	T		D	A	D × A
Total bacteria (log copy/1 ml of rumen content)	8.22	8.07	8.24	7.91	8.21	8.11	8.12	8.09	8.14	8.30	0.05	0.17	0.34	0.02
**Proportion (% of total bacteria)**														
Fibrobacter succinogenes	0.02	0.02	0.17	0.40	0.42	0.26	0.20	0.04	0.05	0.01	0.07	0.73	<0.01	0.28
Ruminococcus flavefaciens	1.55	0.47	0.56	0.38	0.54	0.51	0.35	0.80	0.43	0.74	0.18	0.59	0.33	0.07
Ruminococcus albus	0.02	0.01	0.02	0.11	0.15	0.12	0.05	0.03	0.02	0.02	0.02	0.84	0.01	0.51
Butyrivibrio spp.	1.47	7.42	8.06	7.02	5.44	6.68	4.95	5.79	3.34	5.61	0.89	0.09	0.11	0.13
Prevotella spp.	7.57^{c}	37.70^{ab}	39.70^{ab}	48.81^{a}	30.14^{ab}	23.98^{bc}	24.48^{bc}	23.26^{bc}	21.69^{bc}	27.93^{ac}	3.57	0.05	<0.01	<0.01
Selenomonas ruminantium	0.01^{b}	0.74^{a}	0.35^{ab}	0.46^{ab}	0.13^{ab}	0.39^{ab}	0.38^{ab}	0.53^{ab}	0.69^{a}	0.76^{a}	0.09	0.01	0.02	0.16
Megasphaera elsdenii	2.28	0.95	0.84	1.47	0.38	0.42	0.97	0.93	2.48	2.23	0.48	0.69	0.10	0.80
Treponema spp.	0.01	0.10	0.06	0.07	0.04	0.03	0.05	0.02	0.02	0.03	0.02	0.44	0.51	0.16
Streptococcus bovis	0.10^{b}	1.79^{a}	0.01^{b}	<0.01^{b}	<0.01^{b}	<0.01^{b}	<0.01^{b}	<0.01^{b}	<0.01^{b}	<0.01^{b}	0.15	0.01	<0.01	<0.01
Anaerovibrio lipolytica	<0.01	<0.01	<0.01	<0.01	<0.01	<0.01	0.01	0.01	0.01	<0.01	<0.01	0.23	<0.01	0.47
Ruminobacter amylophilus	<0.01	<0.01	<0.01	<0.01	<0.01	<0.01	<0.01	<0.01	<0.01	<0.01	<0.01	0.07	0.14	0.06

Values are shown as the mean of relative abundance (% of total reads).

C: control group.

T: treatment group.

*Calves in the treatment group were offered fibrous diet including timothy hay and psyllium (Kodithuwakku et al., 2021) via oral administration from 3 days of age until weaning in addition to the voluntary intake of timothy hay.

#*P*-values of the fixed effect of D (Dietary group), A (age) and D × A interaction was calculated by the repeated measure model.

a-cMean values with different superscripts differ ($P < 0.05$), for the treatment.

Supplementary Table S7. Long-lasting effect of oral fiber administration* to calves on the rumen bacteria at 9 months of age and around calving determined by quantitative real-time PCR

Bacterial species/group	Age†										SEM	*P* -value#		
	9 months		-60		-21		0		+21					
	C	T	C	T	C	T	C	T	C	T		D	A	D × A
Total bacteria (log copy/1 ml of rumen content)	7.57	7.54	7.75	7.72	7.81	7.81	7.76	7.66	7.71	7.86	0.04	0.64	<0.01	0.26
**Proportion (% of total bacteria)**														
Fibrobacter succinogenes	0.01	0.01	0.04	0.04	0.07	0.05	0.07	0.11	0.06	0.07	0.01	0.49	<0.01	0.56
Ruminococcus flavefaciens	0.19	0.23	0.21	0.33	0.17	0.28	0.06	0.12	0.07	0.07	0.03	0.06	<0.01	0.69
Ruminococcus albus	0.04	0.02	0.01	0.02	0.02	0.01	0.02	<0.01	0.01	0.01	<0.01	0.09	<0.01	0.04
Butyrivibrio spp.	7.21	6.92	6.68	6.05	6.22	7.26	8.86	9.71	9.66	6.98	0.60	0.55	0.01	0.31
Prevotella spp.	26.56	17.47	27.73	23.93	28.26	18.14	28.52	36.79	32.28	21.79	2.27	0.07	0.01	0.03
Selenomonas ruminantium	0.58	0.30	0.16	0.28	0.26	0.13	0.11	0.38	0.23	0.26	0.06	1.00	0.02	0.01
Megasphaera elsdenii	<0.01	<0.01	<0.01	<0.01	<0.01	<0.01	<0.01	<0.01	<0.01	<0.01	<0.01	0.29	0.01	0.07
Treponema spp.	0.07	0.04	0.03	0.02	0.04	0.02	0.07	0.10	0.06	0.09	0.01	0.78	<0.01	0.07
Streptococcus bovis	0.34	0.29	0.46	0.18	0.15	0.13	0.02	0.07	0.02	0.01	0.06	0.24	<0.01	0.31
Anaerovibrio lipolytica	0.01	0.01	0.01	0.01	0.01	<0.01	0.01	0.02	0.01	0.01	<0.01	0.37	0.03	0.01
Ruminobacter amylophilus	<0.01	<0.01	<0.01	<0.01	<0.01	<0.01	<0.01	<0.01	<0.01	<0.01	<0.01	0.26	0.72	0.79

Values are shown as the mean of relative abundance (% of total reads).

C: control group.

T: treatment group.

*Calves in the treatment group were offered fibrous diet including timothy hay and psyllium (Kodithuwakku et al., 2021) via oral administration from 3 days of age until weaning in addition to the voluntary intake of timothy hay.

†Days relative to calving except for 9 months of age.

#*P*-values of the fixed effect of D (Dietary group), A (age) and D × A interaction was calculated by the repeated measure model.

Supplementary Table S8. Dietary components and chemical composition of the dietary ingredients fed to animals at 9 months of age and around calving

Item	Age*				
	9 months	-60	-21	0	+21
Dietary component (kg/day, as fed)					
Timothy hay	4.0	4.8	7.2	7.2	2.0
Alfalfa hay	2.6	3.2	0.0	0.0	3.0
Fescue hay	0.0	4.0	0.0	0.0	0.0
Oat hay	0.0	0.0	2.4	2.4	4.0
Concentrate	1.6	0.6	1.8	1.8	7.2
Soybean meal	0.0	0.0	0.0	0.0	3.4
Beet pulp	0.0	0.0	0.0	0.0	5.0
Wheat bran	0.0	0.0	0.0	0.0	3.5
Ingredient (% dry matter)					
Crude protein	9.4	8.2	11.3	11.3	14.7
Ether extract	2.1	1.7	2.1	2.1	4.4
Neutral detergent fiber	47.7	59.5	51.2	51.2	33.9
Starch	13.1	3.7	7.1	7.1	19.1
Ash	5.7	5.8	5.8	5.8	8.5

*Days relative to calving except for 9 months of age.

Supplementary Table S9. Primers for quantitative real-time PCR used in this study and standard curves[1] obtained from the amplification profile

	Primers used		Standard curves obtained		
Target	Primer sequences (5´-3´)	Reference	Slope	Y intercept	Efficiency
Total bacteria	[F] CCTACGGGAGGCAGCAG<br>[R] ATTACCGCGGCTGCTGG	Muyzer et al., 1993	-3.047	36.36	2.129
Fibrobacter succinogenes	[F] GGTATGGGATGAGCTTGC<br>[R] GCCTGCCCCTGAACTATC	Tajima et al., 2001	-3.282	35.74	2.017
Ruminococcus flavefaciens	[F] TCTGGAAACGGATGGTA<br>[R] CCTTTAAGACAGGAGTTTACAA	Koike and Kobayashi, 2001	-3.719	39.65	1.857
Ruminococcus albus	[F] CCCTAAAAGCAGTCTTAGTTCG<br>[R] CCTCCTTGCGGTTAGAACA	Koike and Kobayashi, 2001	-3.195	37.56	2.056
Butyrivibrio spp.	[F] GYGAAGAAGTATTTCGGTAT<br>[R] CCAACACCTAGTATTCATC	Boeckaert et al., 2008	-3.295	35.41	2.01
Prevotella spp.	[F] CACRGTAAACGATGGATGCC<br>[R] GGTCGGGTTGCAGACC	Matsuki et al., 2002	-3.304	36.52	2.008
Selenomonas ruminantium	[F] TGCTAATACCGAATGTTG<br>[R] TCCTGCACTCAAGAAAGA	Tajima et al., 2001	-3.454	38.19	1.948
Megasphaera elsdenii	[F] GACCGAAACTGCGATGCTAGA<br>[R] CGCCTCAGCGTCAGTTGTC	Ouwerkerk et al., 2002	-3.457	39.91	1.946
Treponema spp.	[F] GGCAGCAGCTAAGAATATTCC<br>[R] CCGTCAATTCCTTTGAGTTT	Bekele et al., 2011	-2.908	32.19	2.208
Streptococcus bovis	[F] CTAATACCGCATAACAGCAT<br>[R] AGAAACTTCCTATCTCTAGG	Tajima et al., 2001	-3.858	41.58	1.816
Anaerovibrio lipolytica	[F] TGGGTGTTAGAAATGGATTC<br>[R] CTCTCCTGCACTCAAGAATT	Tajima et al., 2001	-3.545	37.61	1.915
Ruminobacter amylophilus	[F] CAACCAGTCGCATTCAGA<br>[R] CACTACTCATGGCAACAT	Tajima et al., 2001	-3.882	41.10	1.810

[F]: Forward primer, [R]: Reverse primer.
[1]Standard curve was generated by plotting the cycle threshold (Y axis) values against the log copy numbers (X axis).

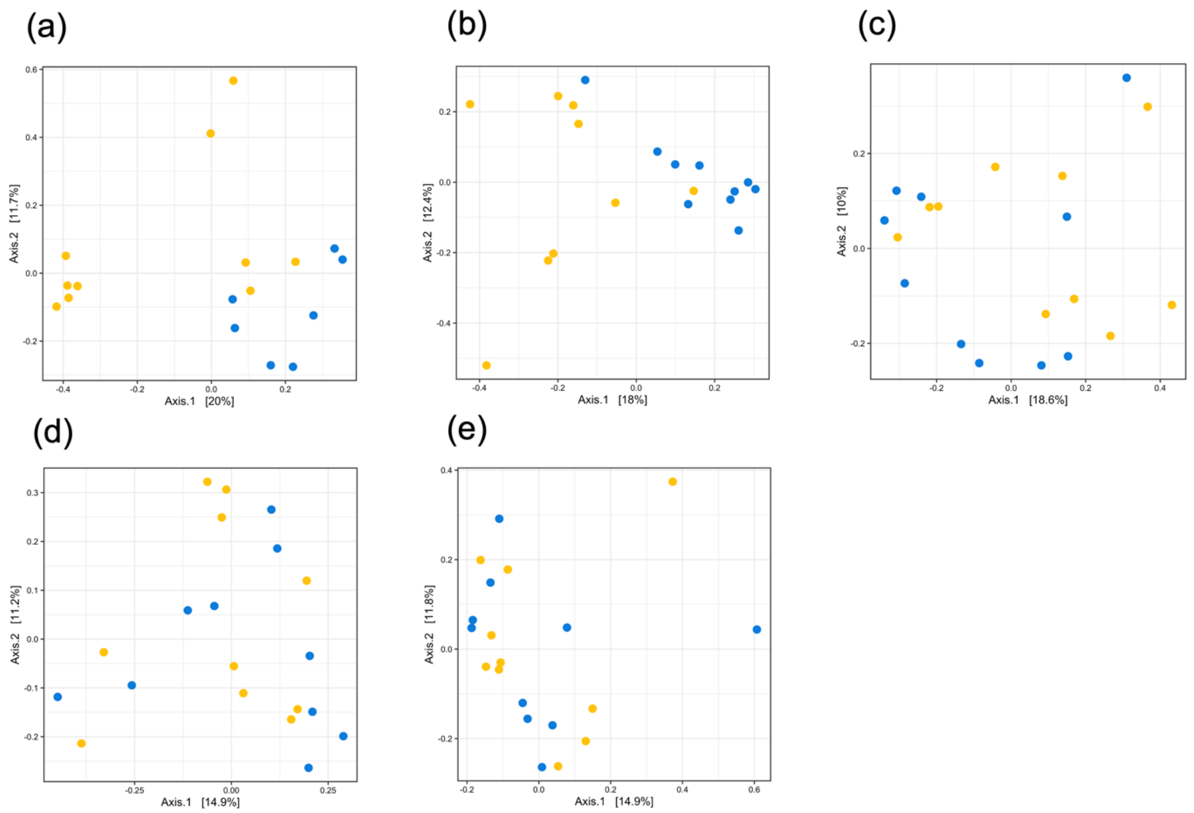

Supplementary Figure S1. Change in the ruminal bacterial community structure in calves. The principal coordinate analysis (PCoA) plot was generated based on Bray-Curtis dissimilarities in rumen bacterial communities determined via 16S rRNA gene amplicon sequencing for respective ages; (a) 7 days, (b) 21 days, (c) 35 days, (d) 49 days, and (e) 56 days. Individual points in each plot represent individual animals. Colors indicate the dietary groups; control (yellow) and treatment (blue). *P*-values of the diet effect at 7 days ($P = 0.005$), 21 days ($P < 0.001$), 35 days ($P = 0.05$), 49 days ($P = 0.15$), and 56 days ($P = 0.26$) were calculated using permutational multivariate analysis of variance (PERMANOVA) test.

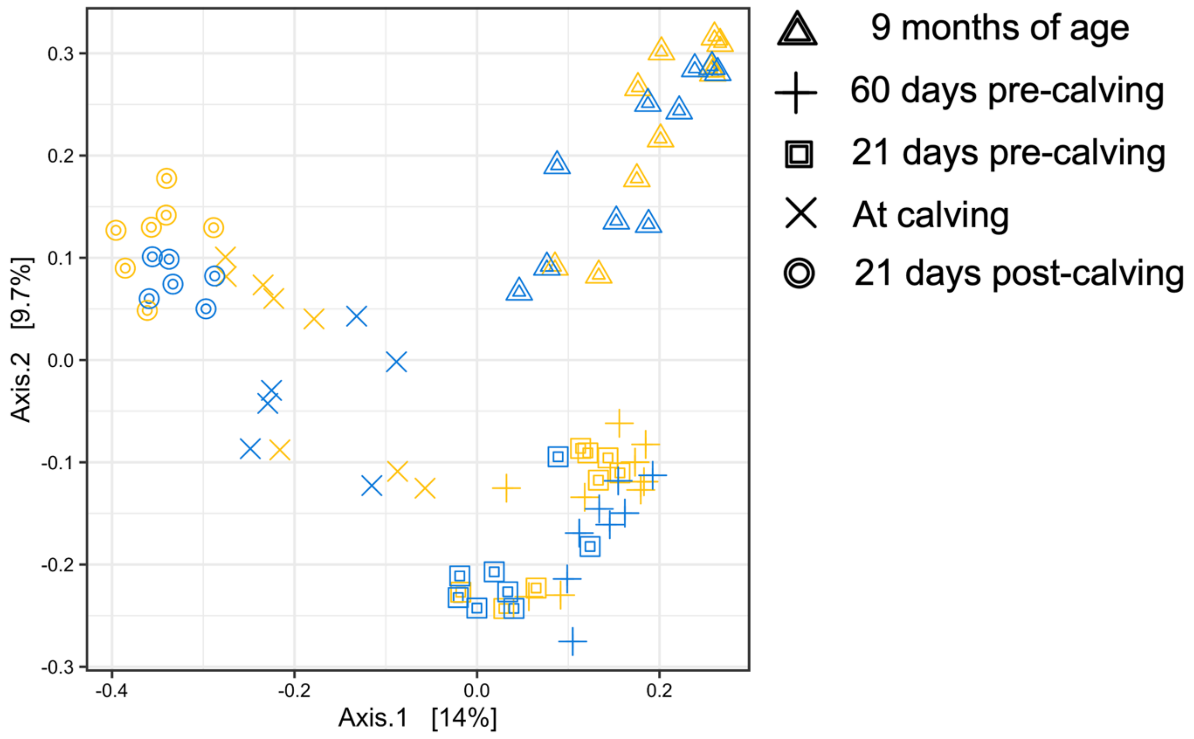

Supplementary Figure S2. Change in the ruminal bacterial community structure in adult cows at 9 months of age and around calving. The principal coordinate analysis (PCoA) plot was generated based on Bray-Curtis dissimilarities of rumen bacterial community determined via 16S rRNA gene amplicon sequencing. Colors indicate the dietary groups; control (yellow) and treatment (blue). Different symbols represent different age points. Individual points represent individual animals. *P*-values of the effect of dietary group ($P = 0.01$), animal age ($P < 0.001$), and diet × age interaction ($P = 0.07$) was calculated using the permutational multivariate analysis of variance (PERMANOVA) test.

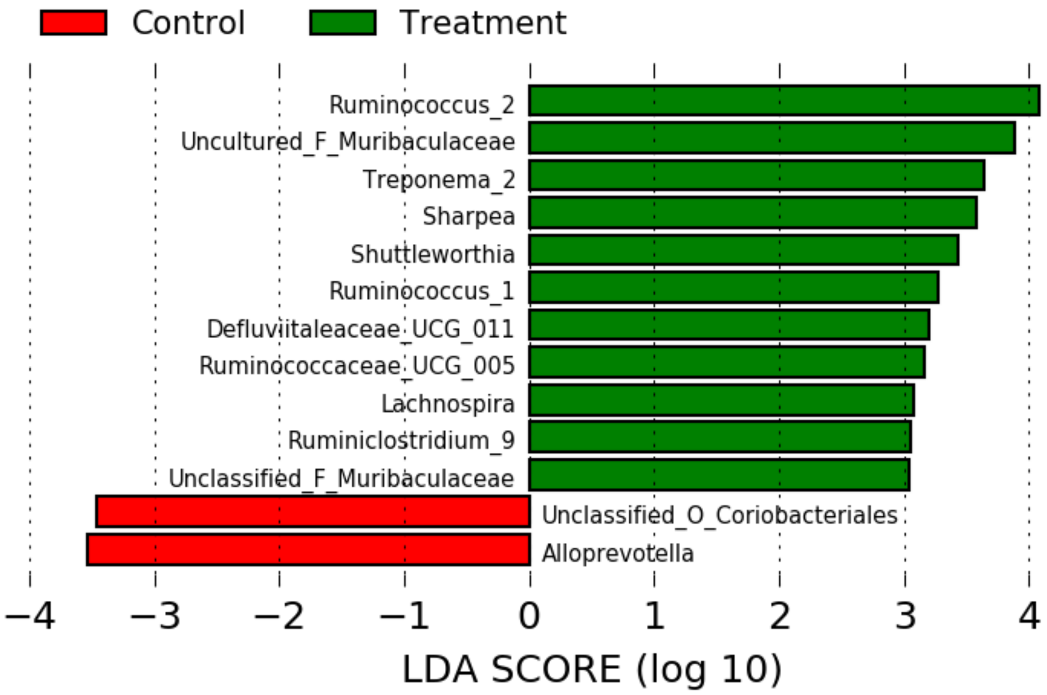

Supplementary Figure S3. Differentially abundant bacterial taxa associated either in control or treatment cows at 21 days after caving. The linear discriminant analysis (LDA) effect size (LEfSe) analysis was performed online in the Galaxy workflow framework (https://huttenhower.sph.harvard.edu/galaxy/). LDA score value of 2.0 was chosen as the threshold for discriminative taxa.

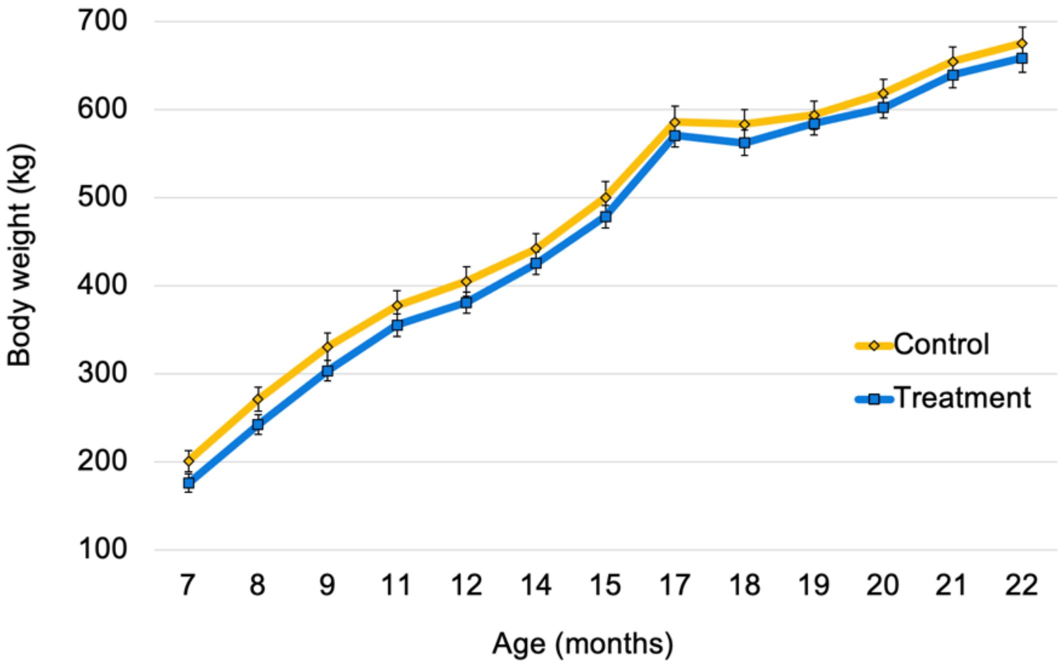

Supplementary Figure S4. Change of the body weight of animals during the growing period. Error bars represent SEM.

References

1. Kodithuwakku, K. A. H. T. *et al.* Effects of oral administration of timothy hay and psyllium on the growth performance and fecal microbiota of preweaning calves. *J. Dairy Sci.* **104**, 12472–12485 (2021).
2. Muyzer, G., de Waal, E. C. & Uitterlinden, A. G. Profiling of complex microbial populations by denaturing gradient gel electrophoresis analysis of polymerase chain reaction-amplified genes coding for 16S rRNA. *Appl. Environ. Microbiol.* **59**, 695–700 (1993).
3. Tajima, K. *et al.* Diet-Dependent Shifts in the Bacterial Population of the Rumen Revealed with Real-Time PCR. *Appl. Environ. Microbiol.* **67**, 2766–2774 (2001).
4. Koike, S. & Kobayashi, Y. Development and use of competitive PCR assays for the rumen cellulolytic bacteria: Fibrobacter succinogenes, Ruminococcus albus and Ruminococcus flavefaciens. *FEMS Microbiol. Lett.* **204**, 361–366 (2001).
5. Boeckaert, C. *et al.* Accumulation of trans C18:1 fatty acids in the rumen after dietary algal supplementation is associated with changes in the Butyrivibrio community. *Appl. Environ. Microbiol.* **74**, 6923–6930 (2008).
6. Matsuki, T. *et al.* Development of 16S rRNA-gene-targeted group-specific primers for the detection and identification of predominant bacteria in human feces. *Appl. Environ. Microbiol.* **68**, 5445–5451 (2002).
7. Ouwerkerk, D., Klieve, A. V. & Forster, R. J. Enumeration of Megasphaera elsdenii in rumen contents by real-time Taq nuclease assay. *J. Appl. Microbiol.* **92**, 753–758 (2002).
8. Bekele, A. Z., Koike, S. & Kobayashi, Y. Phylogenetic diversity and dietary association of rumen Treponema revealed using group-specific 16S rRNA gene-based analysis. *FEMS Microbiol. Lett.* **316**, 51–60 (2011).
